# Identifying priority habitat for conservation and management of Australian humpback dolphins within a marine protected area

**DOI:** 10.1038/s41598-020-69863-6

**Published:** 2020-09-01

**Authors:** Tim N. Hunt, Simon J. Allen, Lars Bejder, Guido J. Parra

**Affiliations:** 1 Cetacean Ecology, Behaviour and Evolution Lab, College of Science and Engineering, Flinders University, Sturt Road, Adelaide, SA 5042 Australia; 2 School of Biological Sciences, University of Western Australia, Stirling Highway, Perth, WA 6109 Australia; 3 School of Biological Sciences, University of Bristol, Tyndall Avenue, Bristol, BS8 1TQ UK; 4 Department of Anthropology, University of Zurich, Rämistrasse 71, 8006 Zurich, Switzerland; 5 Aquatic Megafauna Research Unit, Centre for Sustainable Aquatic Ecosystems, Harry Butler Institute, Murdoch University, South Street, Perth, WA 6150 Australia; 6 Marine Mammal Research Program, Hawaii Institute of Marine Biology, University of Hawaii at Manoa, Manoa, HI 96734 USA

**Keywords:** Behavioural ecology, Ecological modelling

## Abstract

Increasing human activity along the coast has amplified the extinction risk of inshore delphinids. Informed selection and prioritisation of areas for the conservation of inshore delphinids requires a comprehensive understanding of their distribution and habitat use. In this study, we applied an ensemble species distribution modelling approach, combining results of six modelling algorithms to identify areas of high probability of occurrence of the globally Vulnerable Australian humpback dolphin in northern Ningaloo Marine Park (NMP), north-western Australia. Model outputs were based on sighting data collected during systematic, boat-based surveys between 2013 and 2015, and in relation to various ecogeographic variables. Water depth and distance to coast were identified as the most important variables influencing dolphin presence, with dolphins showing a preference for shallow waters (5–15 m) less than 2 km from the coast. Areas of high probability (> 0.6) of dolphin occurrence were primarily (90%) in multiple use areas where extractive human activities are permitted, and were poorly represented in sanctuary (no-take) zones. This spatial mismatch emphasises the need to reassess for future spatial planning and marine park management plan reviews for NMP. Shallow, coastal waters identified here should be considered priority areas for the conservation of this Vulnerable species.

## Introduction

Coastal marine environments have been ranked as most heavily impacted from anthropogenic activities^1^. As a result, wildlife that forage, breed, reside or migrate along the coast, particularly species that are long-lived, late-maturing and slow-reproducing, are becoming increasingly endangered^2–4^. Small odontocetes found in coastal and riverine habitats are examples of such vulnerability, with several species currently under threat^5–7^ and others already extinct^8^ or on the brink of extinction^9^ as a direct or indirect result of human activities. Marine protected areas (MPAs) can be effective tools for conserving such taxa, particularly if MPA zoning includes large no-take areas (i.e. areas closed to extractive activities) of suitable habitat^10,11^. Considering the vulnerability of small odontocetes and their role as umbrella species, the protection of important habitat is key for their conservation, and has the potential to contribute towards the broader conservation of biodiversity and support the delineation of no-take zones within MPAs^12,13^. However, ensuring the effectiveness of such protected areas requires a comprehensive understanding of species distribution and habitat relationships therein^14,15^, which is lacking for several existing protected areas^16^.

The lack of spatially explicit information on species distributions and habitat preferences can compromise their effective protection, even when they occur within designated MPAs^17–21^. Although the implementation of MPAs has grown exponentially since the 1960s^22^; only a small proportion contain no-take zones, and, overall, the global tendency is for MPAs to be located in remote areas or those unpromising for extractive activities, leading to the questioning of their effectiveness for conservation^23,24^. When referring to ‘effectiveness’ of MPAs in the context of cetacean protection, we mean those MPAs that explicitly consider cetaceans in their conservation planning and have relevant, measurable objectives that address conservation and restoration of these types of ‘natural capital’ (concept reviewed in^25^; see also^26^). The north-west marine region of Western Australia (WA) is home to several protected marine megafauna species and Australia's largest fringing reef in Ningaloo Marine Park (NMP). The NMP is a multiple-use MPA and part of the Ningaloo Coast World Heritage Area, proclaimed based on its exceptional marine biodiversity and habitat for threatened species, including a myriad of marine megafauna, many of which have been recognised as ‘ecological values’ in the NMP management plan, each with defined management objectives and performance measures^27,28^. However, our understanding of the distribution and habitat use of most of these species, including the recently described Australian humpback dolphin (*Sousa sahulensis*), remains limited, hampering conservation and management efforts^29^.

The Australian humpback dolphin (hereafter “humpback dolphin”) is endemic to shallow (typically < 30 m) coastal waters of tropical northern Australia and southern Papua New Guinea^30^. Studies in selected areas throughout the Australian range of humpback dolphins indicate that populations are small [typically 50 to 150 individuals, sometimes fewer^31–36^ with limited gene flow^32,37^, and relatively small home ranges (< 300 km^2^; ^32,38^)]. The IUCN Red List of Threatened Species recently listed the Australian humpback dolphin as ‘Vulnerable’ due to the species’ small population sizes and cumulative exposure to human activities^39^.

**Figure 1:**
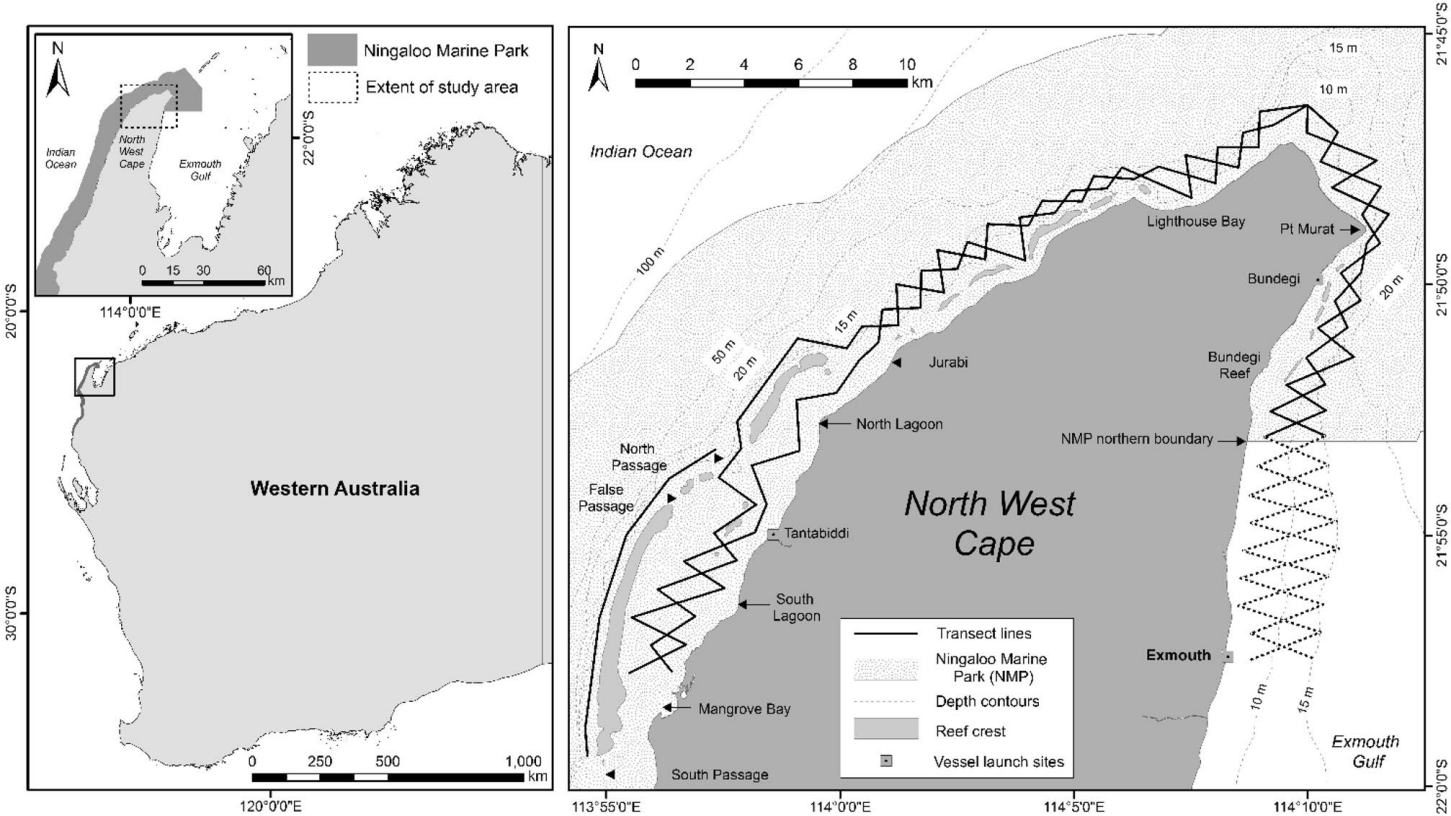
Left: Map of Western Australia, indicating extent of Ningaloo Marine Park, location of North West Cape (NWC), and extent of study area. Right: Map of the NWC study site, including northern Ningaloo Marine Park (NMP) boundary, location names, depth contours, vessel launch sites (Tantabiddi, Bundegi, and Exmouth boat ramps) and opposing zig-zag line transect sampling design. Dotted transect lines indicate the area south of the NMP boundary that were excluded from analyses. Figure created in ArcMap 10.3.1 in ESRI’s ArcGIS© (ESRI, Redlands, California; https://www.esri.com/en-us/arcgis).

MPAs cover a third of the inferred distribution of humpback dolphins in Western Australia, but the efficacy of these reserves in protecting local cetacean populations is unknown^29^. The North West Cape (NWC), located in the northern NMP (Fig. 1), supports the highest density of humpback dolphins (one dolphin per km^2^) recorded to date in Australia^36^. This population (ca. 130 individuals) is characterised by high levels of site fidelity and residency, some seasonality of movement in and out of the study area, and a fission–fusion society displaying assortative interactions by sex and geographic location^36,40^. Despite the apparent importance of this area for humpback dolphins in WA, our understanding of their habitat use is limited. Species distribution models (SDMs, presence-only) for this species based on opportunistic data collected during aerial surveys for dugongs in the western Pilbara region, north and east of the NMP, showed a potential preference for intertidal areas, however, the models were limited by a low sample size and lack of environmental predictor data^41^.

Australian humpback dolphins are a recognised value of MPAs in WA, including the NMP^27^. In light of increasing anthropogenic activities across their range in WA, a better understanding of their distribution and habitat use is needed for robust environmental impact assessments, and the effective implementation and management of protected areas for their conservation^29,42,43^. In this study, we used an ensemble modelling approach to assess the distribution of humpback dolphins within the northern section of the NMP and identify areas of high probability of dolphin occurrence and preferred habitats. Furthermore, we evaluated if the location of current sanctuary zones (i.e. zones where extractive activities like recreational and commercial fishing, and collecting, are not permitted)^27^ is likely to provide protection to humpback dolphins by assessing (1) whether dolphin distribution is correlated with sanctuary zone proximity, and (2) whether or not areas of high dolphin occurrence are encompassed within the boundaries of sanctuary zones.

## Results

**Figure 2:**
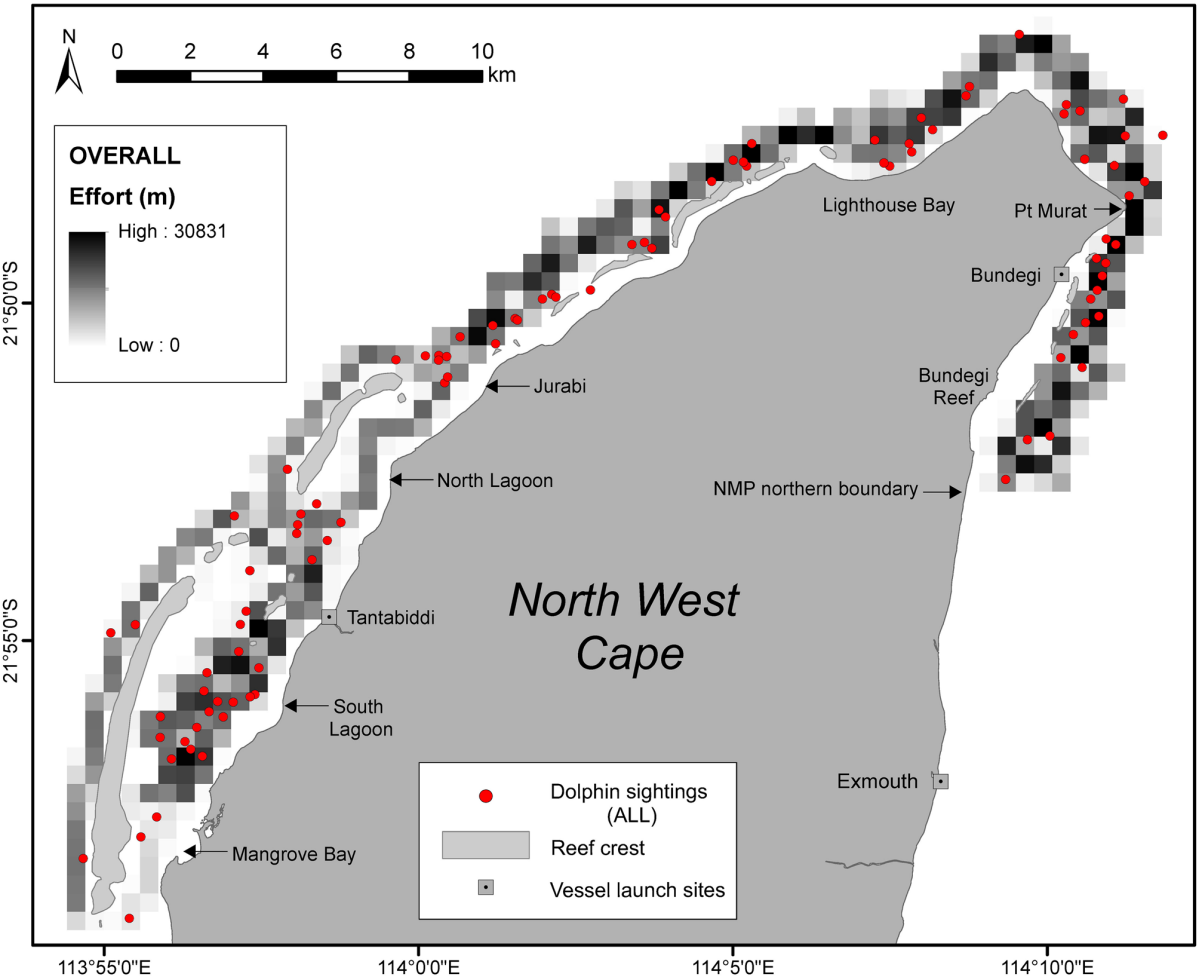
Map of survey effort and sightings of Australian humpback dolphins during boat-based surveys in northern Ningaloo Marine Park (NMP) during the overall survey period, May 2013 to October 2015 (n = 169 sightings). Effort represented as km of survey track lines per 500 × 500 m grid cell. Dolphin sightings represent single or schools of animals. Figure created in ArcMap 10.3.1 in ESRI’s ArcGIS© (ESRI, Redlands, California; https://www.esri.com/en-us/arcgis).

A total of 238 days (or part thereof) of boat-based survey effort, encompassing approximately 330 h and covering 3,627 km of transects in search of dolphins were completed between May 2013 and October 2015 (Table 1, Fig. 2). We encountered 169 humpback dolphin schools over the study period (Table 1, Fig. 2).

**Table 1:** Summary of survey effort, number of dolphin schools encountered and number of 500 × 500 m grid cells with dolphin presences used to model Australian humpback dolphin distribution within northern Ningaloo Marine Park between May 2013 and October 2015.

	Total
Survey days (or part thereof)	238
Survey effort (h)	330
Survey effort (km)	3,627
No. of dolphin schools	169
No. of grid cells with dolphin presences	130

### Model performance

**Figure 3:**
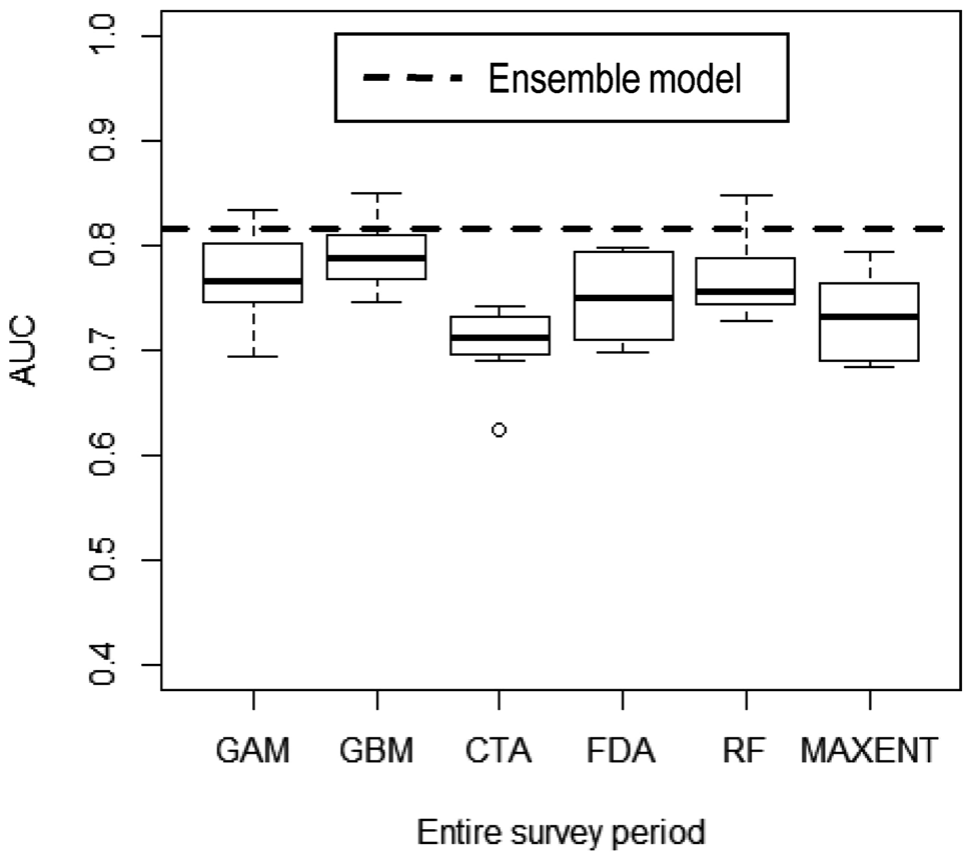
Performance of species distribution models of Australian humpback dolphins in northern Ningaloo Marine Park, Western Australia, built with datasets for the entire survey period (May 2013—October 2015). Box-plot displaying the Area Under Curve (AUC) of the receiver operating characteristics evaluation scores for all models, grouped by modelling algorithm (*GAM *generalised additive model, *GBM* generalised boosted model, *CTA* classification tree analysis, *FDA* flexible discriminant analysis, *RF* random forest, *MAXENT* maximum entropy). Components of box-plot represent minimum (the bottom of the whisker), lower quartile (bottom edge of box), median (bold line drawn inside the box), upper quartile (upper edge of box), maximum (top of the whisker) and outlier AUC values (empty circles) for each modelling method. Dashed line indicates the predictive performance (AUC) of the ensemble model (AUC = 0.82). Values of AUC ≥ 0.7 indicated the ensemble model performed reasonably well.

Collinearity was evident only between distance to reef crest and distance to passage ecogeographic variables (r = 0.9); thus, distance to passage was removed from SDM analysis. Consequently, a total of eight predictor variables were considered in the entire (overall) survey period (Table 2). All single SDMs performed better than random models, and the ensemble model performed better than single models (Fig. 3). The median area under the curve (AUC; see “Methods” for AUC definition) for single SDMs was 0.75, while AUC of the ensemble model was 0.82 (Fig. 3). When considering the mean of means, water depth was the most important variable predicting humpback dolphin distribution (Table 2).

**Table 2:** Importance of ecogeographic predictor variables used in species distribution models (SDMs) of Australian humpback dolphins in northern Ningaloo Marine Park over the entire survey period (May 2013—October 2015).

SDM period	Model	Ecogeographic predictor variables							
		Habitat type	Water depth	Slope	Seabed complexity	Distance to coast	Distance to boat ramp	Distance to reef crest	Distance to sanctuary zone
Entire	GAM	0.179	**0.412**	0.23	0.116	**0.412**	0.051	0.094	0.019
	GBM	0.037	**0.499**	0.129	0.085	0.266	0.02	0.045	0.004
	CTA	0.085	0.556	0.328	0.211	**0.566**	0.074	0.141	0.054
	FDA	0.051	**0.737**	0.108	0.057	0.184	0.000	0.046	0.003
	RF	0.046	**0.239**	0.139	0.091	0.176	0.041	0.055	0.022
	MAXENT	0.069	0.326	0.205	0.108	**0.428**	0.093	0.169	0.097
	Mean of means	0.078	**0.462**	0.190	0.111	0.339	0.047	0.092	0.033

Variable importance is presented as the mean over 10 cross-validation runs of each modelling algorithm, and as the mean of means amongst them.

*GAM* generalised additive model, *GBM* generalised boosted model, *CTA* classification tree analysis, *FDA* flexible discriminant analysis, *RF* random forest, *MAXENT* maximum entropy. Environmental variables of greatest influence based on the randomisation procedure in biomod2 are highlighted in bold. For variable definitions see Supplementary Table S1.1 in Appendix S1.

### Dolphin occurrence across the entire survey period

**Figure 4:**
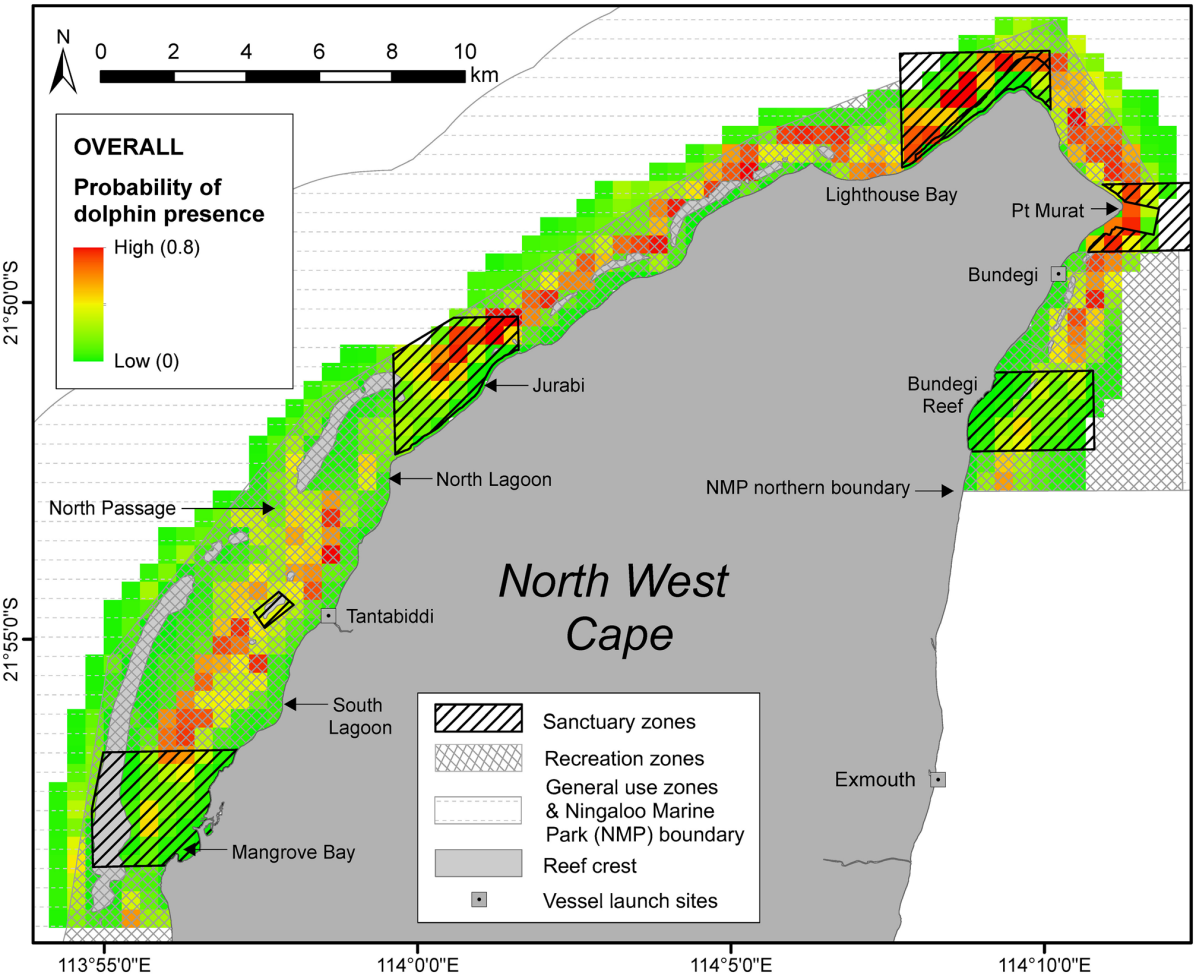
Ensemble model outputs indicating probability of occurrence of Australian humpback dolphins in northern Ningaloo Marine Park (NMP) during the overall survey period, May 2013 to October 2015. Sanctuary zones, recreational zones, general use zones and other locations are also indicated. Figure created in ArcMap 10.3.1 in ESRI’s ArcGIS© (ESRI, Redlands, California; https://www.esri.com/en-us/arcgis).

Across all individual SDMs for the overall dataset, water depth and distance to coast were the two most important ecogeographic variables predicting dolphin occurrence (Table 2). While water depth was the variable with greatest influence overall, individual influence values for this and distance to coast were similar, and across all models were convincingly the strongest predictors of dolphin occurrence (i.e. typically 0.1–0.3 difference between the lesser of these two variables and the third most influential variable; Table 2). Slope and seabed complexity also showed some influence on dolphin distribution (Table 2). The response curves across most individual models indicated that the probability of dolphin occurrence was higher in water depths ranging from 5 to 15 m and less than 2 km from the coast (Supplementary Fig. S2.3 in Appendix S2). Accordingly, the ensemble model predicted high (> 0.6) dolphin presence in shallow waters (mean ± SD = 10.6 ± 4.6; range 4–20 m); within 2 km from the coast between Bundegi Reef in the east and Jurabi in the west, and in the area between North Passage and Tantabiddi, and South Lagoon in the west (Fig. 4). Dolphin occurrence generally increased with increasing slope and seabed complexity (Supplementary Fig. S2.3 in Appendix S2). After depth and distance to coast, benthic habitat was the next most important variable in the generalised additive model (GAM; Table 2), specifically categories of ‘coral reef communities (subtidal)’, sand, and ‘subtidal reef’ (both lagoonal and seaward). For habitat type definitions, see Supplementary Table S1.2 in Appendix S1.

### Dolphin occurrence in sanctuary zones

Sanctuary zones (SZ), or ‘no take’ zones made up 26% of the entire study area, the remainder comprising of recreation zones (60%) and general use zones (14%). For a full list of zone definitions, see Supplementary Table S1.3 in Appendix S1. Distance to SZ was not considered an important variable influencing humpback dolphin occurrence (Table 2). Overall, the probability of dolphin occurrence inside SZ was low (combined mean < 0.3; Table 3, Fig. 4). Dolphin probability of occurrence was generally highest in Jurabi, Lighthouse Bay and Point Murat SZ (mean range = 0.18–0.37; Table 3, Fig. 4). The mean probabilities of dolphin occurrence were higher in these three SZ than outside (outside mean range = 0.14–0.22). SZ only covered a small proportion of areas of high probability (> 0.6) of dolphin occurrence (range 1–11%; Table 4). Randomisation tests indicated that areas of high probability of dolphin occurrence did not occur within SZ more often than would be expected by chance (*P*-value = 0.25).

**Table 3:** Probability of Australian humpback dolphin occurrence in six sanctuary zones of northern Ningaloo Marine Park predicted by ensemble models for the overall survey period (May 2013-October 2015).

			Dolphin occurrence probability
Sanctuary zone	Area (km^2^)	No. of grid cells	Overall (mean ± SD) (median) (range)
Mangrove Bay	11.4	48	0.13 ± 0.13 0.06 0.04—0.58
Tantabiddi	0.5	2	0.32 ± 0.04 0.32 0.28—0.35
Jurabi	7.5	36	0.30 ± 0.23 0.25 0.03—0.73
Lighthouse Bay	7.6	30	0.34 ± 0.27 0.23 0.03—0.76
Point Murat	4.7	9	0.37 ± 0.21 0.33 0.07—0.70
Bundegi Reef	7	32	0.14 ± 0.11 0.14 0.04—0.43
Combined	38.7	157	0.23 ± 0.21 0.15 0.03—0.76
Outside (RZ & GUZ)	111.8	445	0.22 ± 0.20 0.13 0.04—0.74

Values shown indicate mean (± SD), median, and range of occurrence probability for the total number of 500 × 500 m grid cells occupying each sanctuary zone, sanctuary zone grids combined, or grids outside sanctuary zones (i.e. recreation and general use zones; RZ and GUZ, respectively). See Fig. 4 for visual representation of the probability of dolphin occurrence in (and outside) sanctuary zones.

**Table 4:** Summary of Australian humpback dolphin probability of occurrence throughout the entire study area, and six sanctuary zones in northern Ningaloo Marine Park, for the overall survey period, May 2013 to October 2015.

Dolphin occurrence probability	Overall	
	Entire study area (%)	Sanctuary zones (%)
Low (< 0.3)	72	71
Medium (0.31–0.6)	18	17
High (> 0.6)	11	11

Values shown indicate mean (± SD), median, and range of occurrence probability for the total number of 500 × 500 m grid cells occupying each sanctuary zone, sanctuary zone grids combined, or grids outside sanctuary zones (i.e. recreation and general use zones; RZ and GUZ, respectively). See Fig. 4 for visual representation of the probability of dolphin occurrence in (and outside) sanctuary zones.

## Discussion

Ensuring the efficacy of MPAs in protecting mobile marine megafauna requires an understanding of the distribution and habitat preferences of these animals. Our study identified shallow waters (5–15 m), close to the coast (< 2 km) as the areas of highest probability of humpback dolphin occurrence within the northern section of the NMP. Nevertheless, the majority of areas of high probability of dolphin occurrence were located outside SZ. These findings, in combination with the recent and forecast increases in human activities in the marine park (e.g.^44^) suggest that the shallow, inshore areas identified here need prioritisation to better protect this important area for Australian humpback dolphins. We recommend that future spatial planning and marine park management plan reviews consider the preferred habitat areas identified in this study to mitigate potential impacts from increasing human activities for this resident humpback dolphin population.

Our study, which involved survey effort up to 5 km offshore and in depths to 45 m, supports the preference Australian humpback dolphins have for shallow inshore waters. Although sightings occurred in waters toward the offshore survey limits, they were uncommon. In preliminary research at this site, humpback dolphins were encountered at a mean (± SE) of 1 km (± 0.11) from shore, with the majority of schools (95%) in waters < 15 m deep^45^. All sightings reported throughout the adjacent Exmouth Gulf have been in < 20 m depth^41^; Raudino et al. unpub. data; pers. obs.). Elsewhere in WA, humpback dolphins have been observed some 70 km from the mainland coast at the Montebello Islands Marine Park, but close to the shoreline and in shallow water (i.e. < 10 m^46^). In the Northern Territory, humpback dolphins occur within 20 km of major tidal rivers, and as far as 50 km upstream^47^, and along the east coast of Queensland, they occur primarily in waters of < 15 m depth^48–50^. In southern Papua New Guinea (the Kikori Delta), humpback dolphins were sighted in coastal waters of < 12 m water depth^51^. These observations suggest water depth could be a limiting factor for the distribution of this species. Records of humpback dolphins far from the mainland coast are uncommon and likely due to the broad, shallow physiography of the continental shelf, and abundance of shallow reefs, sand flats and continental islands; with dolphins remaining in shallow water and not necessarily far from shore (i.e. mainland or islands^52,53^). Water depth and distance to coast also appear to be strong predictors of the occurrence of other S*ousa* spp., indicating their preference for < 30 m coastal waters (reviewed in^54^; see also summary in^55^).

We note, however, that the majority of boat-based survey effort around Australia, as in this study (see Supplementary Appendix S2), has been concentrated in shallow, coastal waters (e.g.^31,34,49,56^). Thus, we must acknowledge there may be an inherent bias toward distance to coast being a strong predictor variable in this, and other humpback dolphin studies. Nevertheless, the SDMs applied in this study do take survey effort into account, and there have been few confirmed reports of this species in deeper waters (i.e. > 30 m) off the NMP despite multiple years of commercial tour (aerial and vessel platforms) and research operations.

Food availability, predation risk and anthropogenic activities influence delphinid habitat use^57–59^. Australian humpback dolphins feed on a wide variety of fish associated with shallow coastal-estuarine environments^60^, which may explain their preference for shallow coastal waters. Reef structures in the study area are located close to shore and coincide with areas of high dolphin occurrence (e.g. Bundegi Reef to Point Murat, channel from Tantabiddi to North Passage, and South Lagoon). Benthic habitat type including ‘coral reef communities (subtidal)’, and ‘subtidal reef’ (both lagoonal and seaward) showed some importance in our results. In Queensland, humpback dolphins also showed preferences for reef (coral and fringing) habitat type, as well as seagrass flats, mangroves and dredged channels^38,49,50^. The density of herbivorous fish assemblages in Ningaloo Reef, including unicorn fish (*Naso fageni*), were found to be greater around coral reef structures^61^. Humpback dolphins were observed feeding on unicorn fish (*Naso* sp.) in the study area (Hunt, pers. obs.), so these inshore reefs may serve as important foraging areas for this population.

Fish assemblages at SZ in NMP have higher biomass and abundance than at sites where fishing is permitted^62^. It was hypothesised by^36^ that consistent prey availability may be influencing regular use of NMP by humpback dolphins. Future studies into the diet of humpback dolphins in the NMP and how it relates to fish assemblages within SZ are needed to assess their importance to humpback dolphins. This in turn would influence the recommendation to modify SZ spatial extent to better encompass identified areas of high dolphin occurrence (see below).

The prevalence of shark bites on tropical inshore dolphins in the Kimberley region of NW Australia were among the highest recorded^63^, suggesting that predation risk is likely a strong influence on habitat use^57,63^. A number of animals in the study population bear evidence of shark bites (Hunt, unpub. data) and predation risk may be influencing humpback dolphin habitat use in the northern section of the NMP. Prey availability and predator presence are likely factors that influence NWC humpback dolphin social structure^40^, and future studies and modelling approaches involving these potential drivers (or suitable proxies, see below) may better elucidate their influence on humpback dolphin occurrence.

The performance of SDMs is influenced by deficiencies and biases in the ecogeographic variables used to build the models (e.g.^64^). Ideally, species observations and ecogeographic variables, such as benthic habitat type, are measured at the same spatial and temporal resolution. The benthic habitat spatial layer used in this study (i.e.^65^) was developed in 1999 and is currently the only benthic habitat spatial data available for the whole northern NMP. Although benthic habitat was not deemed a primary variable of importance for humpback dolphin distribution, future SDM efforts and spatial zoning would benefit from an updated and validated spatial layer of benthic habitat type.

There were some discrepancies between single model outputs in regard to the relative importance of certain ecogeographic variables on humpback dolphin occurrence. The EM approach we used overcame these predictive uncertainties, with all EMs performing better than single models. To this end, we concur with^66^ in encouraging the use of EM approaches in future studies assessing cetacean distribution and habitat use.

SDMs of marine mammals do not often take into account environmental and behavioural processes that are important drivers of animal distributions, such as prey availability, predation risk, and animal behaviour^67^. This is generally because they are difficult to sample, and do not always offer better model performance. For example,^68^ found that relying on prey distribution data alone was insufficient, and that fine scale models of marine predator habitat selection in coastal habitats will be more successful if environmental variables are used as proxies of both prey and predator distribution. Although behavioural data was collected during the present study, the paucity of data prevented its use for building behaviour-specific models of occurrence (as in^69^), or for using kernel density estimates of behaviours to investigate overlap with areas of high dolphin occurrence (e.g.^66^). Further studies focusing on the collection of focal behavioural data will help address how these behavioural processes influence humpback dolphin distribution in the NMP region.

The almost continuous high areas of occurrence for much of the northern NMP study area corroborate that the NWC is an important habitat for humpback dolphins^36,45^. However, the majority of areas of high probability of dolphin occurrence (> 90%) identified in this study were outside SZ, in recreation zones, where extractive activities such as recreational fishing are allowed. The NMP was initially gazetted in 1987, with the current sanctuary zones gazetted in 2004. The management plan for the NMP (‘the Plan’) has gone beyond its 10-year management period, and, under the *Conservation and Land Management Act 1984*, is due for review “*as soon as possible”*^27^. The forthcoming review represents an opportunity to utilise the adaptive management framework of the Plan to review current or proposed zoning that takes into consideration the areas of high humpback dolphin occurrence identified here in order to minimise disturbance and/or displacement from human activities. The review also represents an opportunity for humpback dolphins to be considered more explicitly with defined management objectives and performance measures^70^.

The areas around Tantabiddi and North Passage are characterised by high probability of dolphin occurrence and have also been identified as part of a core area of very high recreational fishing pressure in NMP^71^. The impact this overlap may have on dolphins is unknown and needs to be assessed. Another location of high dolphin occurrence is the Bundegi/Pt Murat area, which coincides with high recreational boat use^27^, and areas of medium–high dolphin use around North Passage, Tantabiddi, and South Lagoon align with areas of known boat traffic and high recreational use^72^. Given these spatial overlaps and the potential risk of boat strike and/or disturbance to dolphins, consideration should be given to proclaiming ‘go slow’ areas, as adopted in MPAs in Queensland (e.g.^73^) and in Bunbury, WA (see^74^). A more immediate, interim management measure could include the development of educational and interpretive material (e.g. signage at boat ramps, key messages in tourism brochures) highlighting the areas identified as important habitat for humpback dolphins and a recommendation to slow down and adhere to minimum approach distances.

When proclaiming the Ningaloo Coast in 2011, the World Heritage Committee identified that additional management efforts would be required as tourist numbers increased^28^. Given the evidence of increasing human use within the NMP and the conservation value this MPA can provide for future management of this listed Vulnerable species, we recommend that future marine spatial planning reviews reconsider SZ boundaries to better encompass areas of high humpback dolphin occurrence. This is particularly pertinent given one of the objectives of SZ is to provide the highest level of protection for vulnerable species^27^ (Supplementary Table S1.3 in Appendix S1), and that no-take marine reserves have found to be the most effective protected areas in the ocean (reviewed in^75^). It was suggested by^76^ that increases in SZ areas within NMP (e.g. around Bundegi and Jurabi) are needed to better encompass areas critical for resilience to climate change induced disturbance. Increases in SZ areas are also likely to have indirect benefits to humpback dolphins within the MPA through preservation of important habitat as refugia for them and their prey.

## Methods

### Study site

The study site is within the northern section of the NMP, extending from the northern NMP boundary in Exmouth Gulf around the tip of the NWC, and south to Mangrove Bay (inside lagoon) and South Passage (outside reef; Fig. 1). The area is characterised by shallow (< 5 m depth) lagoon waters, with primarily sandy substrate and coral communities within the fringing (sub-tidal) coral reef system^27,77^. Water depth on the western side of the NWC drops sharply outside the reef crest towards the continental shelf, with maximum tidal ranges extending up to 2.5 m.

### Survey design and data collection

Boat-based surveys for humpback dolphins were conducted around the NWC during May–October 2013, April–October 2014 and May–October 2015. Surveys were conducted following a systematic line transect sampling design (2 × 93 km in length, opposing, evenly-spaced zig-zag lines; and 1 × 13 km single line; Fig. 1). Only survey effort and dolphin sighting information collected within the boundaries of the NMP (169 sightings out of 193) was considered for species distribution modelling analyses. The area south of the NMP northern boundary (as indicated by dotted transect lines; Fig. 1) was excluded from analysis because spatial data on benthic habitat is not available for the area outside NMP. The NMP study area equated to systematic line transect lengths of 2 × 68 km opposing zig-zag lines, and 1 × 13 km single line (as indicated by bold line in Fig. 1). The study area covered approximately 150 km^2^ along ca. 50 km of coastline, and extended up to 5 km offshore, encompassing water depths between 1 and 45 m.

Surveys were conducted on board a 5.6 m research vessel powered by a 100 HP outboard motor at speeds of 10–12 km/h and only in good sighting conditions (Beaufort Sea State ≤ 3 and no rain). Survey effort was continuous from 07:00 to 18:00, depending on suitable sighting conditions. A crew of three to five (mode = four) observers searched for dolphins forward of the vessel’s beam with the naked eye and 7 × 50 binoculars. Once a school of dolphins was sighted, search effort was suspended and dolphins were approached to within 10–30 m to record their GPS location, school size, school age composition (calf, juvenile, adult; as defined in^31^), and predominant behaviour (i.e. behavioural state of more than 50% of the animals in a school ^78^). Schools were defined as dolphins with relatively close spatial cohesion (i.e. each member within 100 m of any other member) involved in similar (often the same) behavioural activities (modified from^79^). Although behavioural data was collected, behaviour was not considered as a predictor variable in species distribution analyses (see “Discussion”).

Environmental measurements of water depth and sea surface temperature (SST) were recorded in situ at dolphin sighting locations, at the beginning/end point of transects (n = 87, termed ‘Transect Environmental Station, or ‘TES’), and every 60 min of transect survey effort (termed ‘ES’, see Supplementary Fig. S1.1 in Appendix S1). We used the research vessel’s depth sounder and an Oakton handheld multi-parameter to record water depth and SST, respectively.

### Ecogeographic predictor variables

SDMs aim to predict the spatial distribution of individual species by correlating observations of species occurrence with data on ecogeographic and anthropogenic variables (i.e. predictor variables), thought to influence species distributions. Ecogeographic variables considered in modelling humpback dolphin distribution were either biotic (i.e. benthic habitat type), abiotic (i.e. water depth, slope, seabed complexity, SST, distance to coast, distance to reef crest), or anthropogenic (i.e. distance to SZ, distance to passage, and distance to boat ramp, which were used as a proxy for human activity) (Supplementary Table S1.1 in Appendix S1). Previous research indicates that some of these biotic and abiotic ecogeographic variables likely influence dolphin distribution^80^. Digital environmental layers of water depth and SST were created and explored using environmental data collected in situ at TES, ES and dolphin school sightings (including sightings of Indo-Pacific bottlenose dolphins *Tursiops aduncus*). In deriving digital layers, a mean TES value from each of the 87 fixed locations was obtained for the entire survey period, and by ‘season’ (see below), where *n* per TES ranged from 2 to 30, total *n* = up to 1,582).

Benthic habitat data covering the entire spatial extent of the study area was obtained through the Western Australian Government Parks and Wildlife Service of the Department of Biodiversity, Conservation and Attractions (formerly Department of Parks and Wildlife). This habitat data was derived from the broad scale marine habitat study of the NMP, outlined in^66^. Habitat types within the study area included ‘coral reef communities (subtidal)’, ‘subtidal reef (low relief—seaward)’, ‘subtidal reef (low relief—lagoonal)’, ‘coral reef communities (intertidal or shallow/limestone)’, sand, macroalgae (limestone reef), shoreline reef, salt marsh, mangroves, mudflats, and ‘deep water mixed filter feeding and soft bottom communities’ (for definitions see Supplementary Table S1.2 & Fig. S1.2 in Appendix S1). Water depth across the study area was obtained from hyperspectral imagery (see^81^), then cross-checked and validated using a combination of in situ measurements of water depth (from TES, ES and dolphin sightings, see above, see also Supplementary Fig. S1.1 in Appendix S1), and bathymetric grids from Geoscience Australia^82,83^ (see Supplementary Table S1.1 in Appendix S1).

All ecogeographic variables were sampled at a 500 × 500 m grid resolution using ArcMap 10.3.1 in ESRI’s ArcGIS© (ESRI, Redlands, California) and the Universal Transverse Mercator projection Zone 50 South based on the WGS 1984 datum (Supplementary Fig. S1.3 in Appendix S1). This resolution ensured sufficient detail of each variable throughout the study area, and corresponded with the sampled scale of the dolphin presence-absence data (see below). We used the Spatial Analyst extension in ArcMap 10.3.1 to calculate the Euclidean distance (the shortest straight line distance) for distance to coast, and the Cost distance tool (the shortest distance factoring in land given study area wraps around a peninsula) for distance to reef crest, distance to SZ, distance to boat ramp, and distance to passage (see Supplementary Table S1.1 & Fig. S1.3 in Appendix S1). SST was calculated using the Ordinary Kriging interpolation tool with a spherical semivariogram model (500 m cell size, 12 point variable search radius size) in the Spatial Analyst extension in ArcMap (see Supplementary Table S1.1 in Appendix S1).

### Data exploration

Ecogeographic predictor variables considered for SDMs were grouped for the entire survey period from May 2013 to October 2015 (i.e. an overall SDM, using all fixed predictor variables outlined in Supplementary Table S1.1 in Appendix S1). Surveys were not conducted during the summer period (i.e. November to March), due to the occurrence of strong winds and tropical cyclones. Prior to running the SDMs, collinearity (correlation between environmental variables) was investigated in R v3.3.1^84^ using multi-panel scatterplots, Pearson’s correlation coefficient (r) and variance inflation factors (VIFs) for all combinations of variables in the overall model^85^. Highly correlated variables were identified using the stepwise procedures *vifcor* and *vifstep* in the package *usdm* in R^86^. Using the *vifcor* procedure, whenever the maximum linear correlation between two variables was greater than the threshold (r = 0.7;^85^), that with the highest VIF is excluded; this step was repeated until no variable remained with an r-value greater than the threshold. Similarly, using *vifstep*, the variable with the highest VIF, and greater than the threshold (VIF = 3;^85^), was excluded; this step was also repeated until no variable with a VIF greater than the threshold remained^86^.

### Response variable

The presence-absence of humpback dolphins (schools or single animals) was used as the response variable for ensemble species distribution modelling. The locations of dolphin sightings obtained on survey effort, and the associated survey tracks, were imported into ArcMap, and binary presence-absence grids were prepared. Survey coverage was quantified by adding a 250 m buffer either side of each survey track line, which was the average distance to which dolphins could be reliably observed from the boat under a variety of sea conditions (e.g.^66^). Survey effort was then quantified by intersecting track lines with the 500 × 500 m gridded area of survey coverage and calculating the length of survey effort track (km) per grid cell^87^. Each 500 × 500 m grid cell was classified as either 1 (dolphin presence) or 0 (dolphin absence), and was also characterised by each of the environmental predictor variables (see Supplementary Table S1.1 in Appendix S1).

To reduce false absences in SDMs (i.e. a species is considered absent from an area when it may in fact occur in that area; see^88,89^), absence cells were defined based on areas of highest survey effort^90^. Grid cells within the study area were ranked from highest to lowest effort, and cells with the highest survey effort and no dolphin presence were considered most likely to represent true absences and were thus defined as absence cells (as per^66^). The total number of absence cells was made equal to the total number of presence cells when considering ensemble SDMs. The survey effort threshold (m per grid cell) for defining true absences was 8,727 m for the overall model (highest was 24,274 m).

Most species SDMs are built at a grid cell size of 1 km × 1 km, which has been criticized as being too coarse to generate reliable SDM outputs^91^. This is particularly true for studies at small spatial scales, such as this. Model accuracy is likely to increase with decreasing cell size, but too fine a cell size has been shown to amplify the error from background absences^92^. Given: dolphins are highly mobile; the locational error associated with dolphin locations (i.e. dolphin locations are recorded within 10 to 30 m of research vessel); our definition of school size (i.e. each member within 100 m of any other member); and survey coverage (250 m buffer each side of the survey track line), we considered a 500 m cell size appropriate resolution while maintaining details of the attributes of the study area and scale at which dolphin and environmental data were collected. Reliable outputs based on this cell size have resulted from prior SDM studies of coastal dolphins (e.g.^66,93^).

### Ensemble species distribution modelling

Species-habitat relationships are often investigated using correlative models to predict species distributions by combining known occurrence records with digital layers of ecogeographic variables expected to affect the species’ distribution^94^. SDMs encompass a variety of modelling algorithms with differences in predictive performance, depending on sample size, data structure (e.g. presence-only, presence-absence, presence/pseudo-absence), and the underlying fitted functions^94–96^. Ensemble modelling (EM) is an approach by which single-model predictions are combined^97,98^, yielding a higher level of accuracy and less bias than separate, single models^66,96,99^. EM approaches have been used across terrestrial species (e.g.^100^), and a variety of marine species (e.g.^101–103^), including blue whales (e.g.^104^) and coastal dolphins^66,93,105^.

We used an EM approach implemented in the biomod2 R package^106^ to predict the presence-absence of humpback dolphins with respect to the ecogeographic predictor variables (Supplementary Table S1.1 in Appendix S1). This approach used six different modelling algorithms under three different modelling methods: two regression methods, generalised additive models (GAMs^107^) and generalised boosted models (GBMs^108^); two classification methods, classification tree analysis (CTA^109^) and flexible discriminant analysis (FDA^110^); and two machine learning methods, random forest (RF^111^) and maximum entropy (MAXENT^112^). We selected these modelling algorithms because they are the most commonly used, known to perform well, and provide a sound comparison across a wide range of modelling approaches including regression, classification and machine learning methods^96,106,113,114^.

SDMs were developed using a binomial error distribution and the logit link function. Data for each SDM were split 75%/25% for model calibration and testing, respectively^106^. A total of 60 different statistical models calibrated for the SDM dataset resulted from a tenfold cross validation process. A randomisation procedure in biomod2 based on 10 permutation runs was subsequently implemented to assess the importance of the environmental predictor variables^106^. This procedure is independent of the modelling technique. It calculates the Pearson correlation between the standard predictions (i.e. fitted values) and predictions where one variable has been randomly permutated. If there is a high correlation between the two predictions (i.e. there is little difference between the two predictions), the variable permutated is considered unimportant for the model and vice versa. This procedure was repeated 10 times for each variable independently, and the correlation means were kept for each variable. Subsequently, this allowed direct comparison between models regardless of the modelling method. The mean correlation coefficient was then used to rank the variables from zero to one; where zero indicates the variable has no influence in the model, and one indicates the variable is most influential in the model^106^. A list of all parameters used for running the selected models in biomod2 is shown in Supplementary Appendix S2.

SDMs that utilise presence-absence data are subject to false positives (predicting species occurrence in areas where the species does not occur) or false negatives (failing to predict species presence where the species does occur^98^). To assess SDM predictive performance and compare individual modelling algorithms, we used the area under the curve (AUC) metric of the receiver operating characteristics plot^115^ calculated in R using biomod2. The AUC is a measure of the ratio between the observed presence-absence values and the model predictions. Values range from zero to one, with values above 0.5 indicative of models with predictions performing better than what would be expected by chance^115^. In general, AUC values of 0.5–0.7 are considered low and represent poor model performance, values of 0.7–0.9 are considered reasonable predictions, and values above 0.9 represent excellent model performance^116^.

Lastly, we combined the six individual SDMs (modelling algorithms) to obtain an ensemble prediction of dolphin presence across the study area^106^. Of the individual models, only those with AUC values above 0.5 were considered, and their contribution to the ensemble model was weighted based on their predictive accuracy (the higher the evaluation score the more weight assigned to the model^96^). The ensemble model output was then imported into ArcMap, providing a visual output of probability of species occurrence, where values ranged from zero to one; zero indicating no probability and one indicating a very high probability of dolphin presence. Finally, following^96^, we used AUC values to compare the performance of the ensemble model with the performance of the individual models.

### Dolphin occurrence and sanctuary zones

To evaluate the relevance of the six current SZ in the northern NMP for the protection of humpback dolphins, we assessed whether areas of high dolphin occurrence (i.e. > 0.6) fell within SZ more often than would be expected by chance using a randomisation test in PopTools v3.2.5^117^. To do this, we calculated an observed index for the ensemble output (i.e. total number of high dolphin occurrence cells that were located within SZ) and compared this index with a random index (i.e. total number of times high dolphin occurrence cells fell within SZ as they were randomly distributed across the study area), obtained from 5,000 permutations. The significance (*P*-value ≥ 0.05) was calculated as the proportion the random index that was greater than or equal to the observed index^118^.

### Seasonality of dolphin occurrence

Demographic analysis from^36^ indicated that there was some seasonality of humpback dolphin movement in and out of the study area. Using the above methodology, models were also split temporally into corresponding seasons (i.e. Autumn–Winter, April to July inclusive; and Winter-Spring, August to October inclusive) to determine if these demographic characteristics are reflected in changes in the probability of occurrence and habitat preferences. The ensemble models show consistent results in the spatial distribution of humpback dolphins among seasons (see Supplementary Appendix S3 for further details on seasonal analysis), and thus we conducted further analysis on the pooled dataset.

### Approvals

Data collection was permitted by the Western Australian (WA) Government Parks and Wildlife Service of the Department of Biodiversity, Conservation and Attractions (SF009240, SF009768, SF010289), WA Government Agriculture and Food Division (U38/2013-2015) and the Australian Government Department of Defence (Harold Holt Naval Base Exmouth), with approval from Flinders University Animal Welfare Committee (project number E383).

## Supplementary information

Supplementary Information.

## Data Availability

Data made available to all interested researchers upon reasonable request to Tim Hunt (t_hunt@live.com.au) and Guido J. Parra (guido.parra@flinders.edu.au).
