# Supplementary material for: Identifying priority habitat for conservation and management of Australian humpback dolphins within a marine protected area
Source: Sci Rep. 2020 Sep 1;10:14366. doi: 10.1038/s41598-020-69863-6 (PMC7463025; doi:10.1038/s41598-020-69863-6)
Supplement: Supplementary file 1 — Supplementary Information. [file 41598_2020_69863_MOESM1_ESM.pdf]

Identifying priority habitat for conservation and management of Australian humpback dolphins within a marine protected area

Tim N. Hunt*[1], Simon J. Allen[2,3,4], Lars Bejder[5,6] and Guido J. Parra[1]

SUPPLEMENTARY MATERIAL

[1] Cetacean Ecology, Behaviour and Evolution Lab, College of Science and Engineering, Flinders University, Sturt Road, Adelaide, SA 5042, Australia

[2] School of Biological Sciences, University of Western Australia, Stirling Highway, Perth, WA 6109, Australia

[3] School of Biological Sciences, University of Bristol, Tyndall Avenue, Bristol, BS8 1TQ, United Kingdom.

[4] Department of Anthropology, University of Zurich, Rämistrasse 71, CH-8006, Zurich, Switzerland.

[5] Aquatic Megafauna Research Unit, Centre for Sustainable Aquatic Ecosystems, Harry Butler Institute, Murdoch University, South Street, Perth, WA 6150, Australia

[6] Marine Mammal Research Program, Hawaii Institute of Marine Biology, University of Hawaii at Manoa, HI 96734, USA

* Corresponding author: tim.hunt@flinders.edu.au; t_hunt@live.com.au

Running title: Priority habitat for Australian humpback dolphin conservation

APPENDIX S1

Table S1.1. Descriptive summary of ecogeographic predictor variables used in ensemble species distribution modelling of Australian humpback dolphins in northern Ningaloo Marine Park. All predictor variable raster layers were derived in ArcMap 10.3.1 (ESRI, Redlands, California), and all layers (except habitat type, and depth) used tools in the Spatial Analyst extension*. All predictor variables, excluding SST, were considered fixed (i.e. did not vary temporally). SST was used in seasonal analysis for predictions of dolphin occurrence, see APPENDIX S3.

Predictor variable	Variable type	Variable abbreviation	Description
Benthic habitat type	Biotic (fixed)	Habitat	Broad scale habitat types (from Bancroft and Sheridan (2000) within the study area include: coral reef communities (intertidal or shallow/limestone) [CRCI]; coral reef communities (subtidal) [CRCS]; sand [S], subtidal reef (low relief - lagoonal) [SRL]; subtidal reef (low relief - seaward) [SRS]; shoreline reef [SHR]; macroalgae (limestone reef) [MA]; salt marsh [SM]; mangrove [MN], mudflat [MU]; and deep water mixed filter feeding and soft bottom communities [DWM]. These habitat maps were obtained using remote sensing imagery (25 m pixels) from Landsat aerial photography maps (sampled 1994) and habitat point data validated in the field (sampled 1999). See Table S1.2 and Fig. S1.2 for definitions.<br><br>Habitat type for each grid cell was defined based on the predominant habitat type (i.e. >50% of grid cell area, or closest largest) when intersected with the Bancroft and Sheridan (2000) habitat layer.<br><br>Predominant habitat types within the study area were CRCS (~40%), SRS (~20%), SRL (~15%), CRCI (~10%), and S (~10%). The remaining habitat area (~5%) was made up of MA and SHR. SM, MN, MU, and DWM were not considered in analyses.
Water depth	Abiotic (fixed)	Depth	Bathymetric data was obtained from hyperspectral image mapping (3.5 m pixel resolution) collected in April 2006 (see Kobryn et al. 2011,2013). Due to the hyperspectral data collection process, accuracy of depth measurements beyond 20 m was questionable. To account for this variability, and validate depths <20 m, we overlayed the hyperspectral depth layer with *in situ* measurements of depth (n = 1467; from TES, ES and dolphin sightings; Fig. S1.1) and bathymetric grids obtained from Geoscience Australia (2008, 2009). Where hyperspectral depth discrepancies were >5 m (i.e. in the >20 m readings), depth values were manually altered to reflect the larger depth value. See Fig. S1.3.<br><br>Given tidal range in the study area is up to 2.5 m, correction of depth data in relation to tidal state was not deemed necessary given: a) 500 m grid cells average depth measurements over a spatial scale where depth variation can be >2.5 m, and b) humpback dolphins grow up to 2.7 m in length (Jefferson et al. 2015) so a (maximum) 2.5 m difference in water depth is likely to be negligible in terms of modelling species distribution.

Slope	Abiotic (fixed)	Slope	Derived from the depth layer* (see MacLeod 2013). See Fig. S1.3.
Seabed complexity	Abiotic (fixed)	Complexity	Derived from calculating the standard deviation of the slope layer* (see MacLeod 2013). See Fig. S1.3.
Distance to coast	Abiotic (fixed)	Dist_coast	Grid cells are assigned a distance to coast value using Euclidean distance*. See Fig. S1.3.
Distance to reef crest	Abiotic (fixed)	Dist_reef	Reef crest refers to coral reef habitat (including coral bommies) that are either exposed or very shallow (<1 m) at low tide, and as such can create a physical barrier to dolphin movement. This digital layer was modified from Smallwood et al. (2012) and the Bancroft and Sheridan (2000) 'Coral reef communities (intertidal or shallow/limestone)' layer, as well as nautical charts and author's knowledge of the study area. The purpose of this layer is to determine if offshore reef has an influence on dolphin distribution. Layer therefore does not include exposed or shallow reef adjacent to the shoreline, as the shoreline itself creates a physical barrier to dolphin movement, depending on the tidal level. Reef crest was intersected with each grid cell, and defined when reef crest encompassed >50% of total grid cell area. Each non-reef crest grid cell was assigned a value based on Cost distance* from the mid-point of the cell to the nearest reef crest cell. See Fig. S1.3.
Distance to sanctuary zone	Anthropogenic (fixed)	Dist_sz	A total of six sanctuary zones (no fishing zones) are within the boundaries of the study area (NB. Sanctuary zone polygons in the study area obtained from Western Australian Government Parks and Wildlife Service (see DPaW DoF 2014). Given fish assemblages at sanctuary zones in NMP have higher biomass and abundance than at sites where fishing is permitted (Fitzpatrick et al. 2015), and it's hypothesised that consistent prey availability may be influencing regular use of NMP by humpback dolphins (Hunt et al. 2017), we are considering the presence of the sanctuary zone and distance from it, together. We are making the assumption that highest density of fish is within the sanctuary zone, and that this reduces as a gradient the further you move away from it. Given humpback dolphins are considered opportunistic feeders (Parra & Jedensjö 2014), and the foraging observations observed in the study area (Hunt, unpublished data), we argue that there is a lack of evidence to suggest that sanctuary zones do not support relevant prey taxa for this species. Exploring this variable as a potential predictor of dolphin distribution is therefore justified.<br><br>Sanctuary zones were intersected with each grid cell, and defined when the sanctuary zone encompassed >50% of the grid cell area. Each grid cell outside a sanctuary zone was assigned a value based on Cost distance* from the mid-point of the cell to the perimeter of the sanctuary zone. A map of the sanctuary zones within the study area can be viewed in Fig. S1.2, see also DPaW DoF (2014). The distance to sanctuary zone gridded layer can be viewed in Fig. S1.3.

Distance to passage	Anthropogenic (fixed)	Dist_passage	North Passage, False Passage, and South Passage are areas of deeper water between reef crest that vessels use to transit through when leaving or returning to Tantabiddi boat ramp (Fig. 1). This variable was considered a proxy for human activity given commercial nature-based tourism (e.g. whaleshark swim-with and humpback whale swim-with tour operations) and recreational fishing vessels use these passages regularly during the tourism season (i.e. March to October) to get seaward side of the fringing reef and out to deeper waters. Points were created at the centre of each passage, and using Cost distance*, calculated the shortest distance from the mid-point of each grid cell to the nearest passage. See Fig. S1.3.
Distance to boat ramp	Anthropogenic (fixed)	Dist_ramp	Tantabiddi and Bundegi boat ramps (Fig. 1) are frequently used by vessels during the tourism season. This anthropogenic variable was also considered a proxy for human activity (i.e. vessel movement). Points were created at each of the two boat ramps, and using Cost distance*, calculated the shortest distance from the mid-point of each grid cell to the nearest boat ramp. See Fig. S1.3.
Sea surface temperature	Abiotic (temporal)	SST	SST was interpolated for both the Autumn-Winter (AW) and Winter-Spring (WS) seasons using the Original Kriging method (spherical semivariogram model with a 500 m output cell size and a 12 point variable search radius)*. The interpolated layer was derived from *in situ* measurements of SST (n = 405 for AW, and 521 for WS) at TES, ES and dolphin sightings (Fig. S1.1). The output variance of prediction raster was also calculated for each interpolated layer. Low values (0.7-3.4) indicated a high degree of confidence in the predicted values. See Fig. S3.2 in APPENDIX S3.

Table S1.2. Benthic habitat category definitions from Bancroft and Sheridan (2000) used in ensemble species distribution modelling (SDM) of Australian humpback dolphin in northern Ningaloo Marine Park. Categories above the bold double black line were included in SDMs.

Habitat code	Habitat category	Definition (from Bancroft and Sheridan 2000)
CRCI	Coral reef communities (intertidal or shallow/limestone)	Located in the intertidal or shallow regions (<1 m LAT) on a limestone substrate. This habitat includes the reef crest, reef flats and shallow back reef zones. Live coral cover varies greatly and some areas have a high proportion of coral rubble. Macroalgae, sand or pavement also may be present. Hard corals (e.g. *Acropora* spp.), soft corals (e.g. *Sinularia* spp.) are typical of the fauna present in these habitats. Parts of this habitat typically support a high diversity and abundance of fish and invertebrate fauna.
CRCS	Coral reef communities (subtidal)	High live coral cover with macroalgal turf and coralline algae covering areas of reef not occupied by living corals. Sand patches, bare pavement and rubble may also be present. This habitat is used to describe the upper seaward slope, sheltered back reef, deep lagoonal reef and bommie clusters. Areas of high coral cover are generally restricted to water depth less than 15 m. Offshore, these habitats are dominated by faster growing coral species such as *Acropora* (hard, branching) and *Montipora*. In the lagoons, coral communities consist of a mixture of *Acropora* and a diverse range of massive species (in particular family *Faviidae.*
S	Sand	Habitat is defined as subtidal habitats that have predominately white carbonate sands as a substrate, however the sand may overlay reef platform or have patches of other habitats present. Sand habitats are typically bare, and may have seasonal vegetation or permanent patches of seagrass or macroalgae. Invertebrate fauna may also be present.
SRL	Subtidal reef (low relief - lagoonal)	Describes subtidal areas of limestone substratum that may incorporate sand patches, rubble and scattered isolated corals. This habitat typically is pavement, which may have low relief (<1 m high) and occurs within the sheltered shallow waters (<10 depth) of the lagoons of the Ningaloo Marine Park. This habitat may support a diverse array of algae and sessile invertebrates including sponges, sea-whips and sea-pens, and may also support some macroalgae (e.g. *Turbinaria* sp., *Sargassum* sp., *Halimeda* sp.), or seagrass (e.g. *Halophila* sp.) in patchy mobile sands. Dugongs (*Dugong dugon*) are often seen feeding in this habitat within Ningaloo Marine Park.
SRS	Subtidal reef (low relief - seaward)	Habitat describes subtidal areas of limestone substratum that may be predominantly covered by sand. This habitat is typically pavement, which may have low relief (< 1 m high) and occurs in the more exposed deeper waters (>15 m depth) seaward of the barrier reef system. This habitat is typical bare or overlaid with large sand patches, however macroalgal turf and sessile invertebrates may also be present.
SHR	Shoreline reef	The shoreline reef habitat is typically located in the lower intertidal or nearshore subtidal zones (<1 m below LAT) and occurs as low relief reef platforms of sedimentary (limestone or sandstone) substratum that are contiguous with the shoreline. In the Ningaloo Marine Park, shoreline reef habitat typically supports turf algae and invertebrates such as molluscs

		(*Tridacna* spp. clams, *Cypraea* sp. cowries), hermit crabs (*Dardanus* sp.) and isolated soft and hard coral communities.
MA	Macroalgae (limestone reef)	Areas of subtidal limestone substratum of low or high relief. In the Ningaloo Marine Park and the proposed southern extension, this habitat is found in shallower waters (<10 m depth) and may also incorporate mobile sand patches, and scattered isolated hard and soft corals. This habitat is generally covered in large fleshy macroalgae (e.g. *Sargassum* spp.) or macroalgal turf (red, green and brown algae). A wide range of invertebrate life such as sponges, ascidians and soft corals, are associated with this habitat.
DWM	Deep water mixed filter feeding and soft bottom communities/ Pelagic	This category is specific to those areas that are greater than 50m in depth. The focus in this classification is not on substrates but rather on the macrobiology of the water column, hence pelagic environments may have various substrates. Pelagic fish and invertebrates, and larval stages of various phyla dominate the macrobiology of this habitat. In the Ningaloo Marine Park, whale sharks (*Rhincodon typus*) and manta rays (*Manta birostris*) are known to feed in the surface waters of this habitat.
SM	Salt marsh	Areas of low relief located in the upper intertidal and supratidal (immediately above HAT) zones of low energy coastlines. The substrata consist of muddy or silty terrigenous sediment. Salt marsh habitats often occur landward of mangroves, tidal creeks and estuaries, and typically supports vegetation, but can also occur as unvegetated coastal saline flats. In the Ningaloo Marine Park burrowing crabs (*Uca* sp.) and ghost crabs (*Ocypode* sp.) are found in this habitat.
MU	Mudflat	Located in the lower intertidal zone and generally consists of terrigenous mud or silt sediments. Mudflats occur in areas of low energy and high deposition such as the areas seaward of mangroves. In the Ningaloo Marine Park, mudflat habitats are typically bare of vegetation, but support gastropods (e.g. *Cerinthium* sp.), crabs and invertebrate infauna.
MN	Mangrove	Areas of mangrove forest greater than 0.05 ha and typically is located in the upper intertidal zone. The substratum of this habitat typically comprises of mud and silt, however some mangrove species do occur on intertidal rocky shores. There are two mangrove species, *Avicennia marina* and *Rhizophora stylosa*, which occur in the Ningaloo Marine Park. Mangrove roots provide a substratum for many gastropods (e.g. *Natica, Cerithium, Strombus*) and other invertebrates, such as the mangrove crab (*Scylla serrata*) are often present.

Table S1.3. Definition of zoning arrangements within the northern Ningaloo Marine Park study area, as defined in the '*Management Plan for the Ningaloo Marine Park and Muiron Islands Marine Management Area 2005 – 2015*' (CALM & MPRA 2005).

Type of zone	Definition
Sanctuary	Sanctuary zones in marine parks provide for the maintenance of environmental values and are managed for nature conservation by excluding human activities that are likely to affect the environment adversely. The primary purpose of sanctuary zones is for the protection and conservation of marine biodiversity. They are used to provide the highest level of protection for vulnerable or specially protected species, and to protect representative habitats from human disturbance so that marine life can be seen and studied in an undisturbed state.<br>Specified passive recreational activities consistent with maintaining environmental values may be permitted, but extractive activities, including fishing and traditional fishing and hunting are not. Commercial tourism operations (such as for nature-based tours) will be considered where they do not conflict with other uses and are regulated under the CALM Act.<br>All extractive activities are excluded from sanctuary zones. Passive nature-based tourism, some recreational activities, boating and approved scientific research are permitted.
Recreation	Recreation zones have the primary purpose of providing opportunities for recreational activities, including fishing, for visitors and for commercial tourism operators, where these activities are compatible with the maintenance of the values of the zone. Petroleum drilling and production, commercial fishing, pearling and aquaculture are not permitted in recreation zones.<br>Recreation zones in marine parks provide for conservation and recreation, including recreational fishing where this is compatible with the conservation values. Commercial fishing, pearling and aquaculture are not permitted in these zones.
General use	General use zones in marine parks are those areas of the marine park not included in sanctuary, special purpose or recreation zones. Conservation of natural values is still the priority of general use zones, but activities such as sustainable commercial and recreational fishing, aquaculture, pearling and petroleum exploration and production may be permitted provided they do not compromise the ecological values of the marine park.<br>All areas not zoned as sanctuary or recreation zones are classified as general use zones.

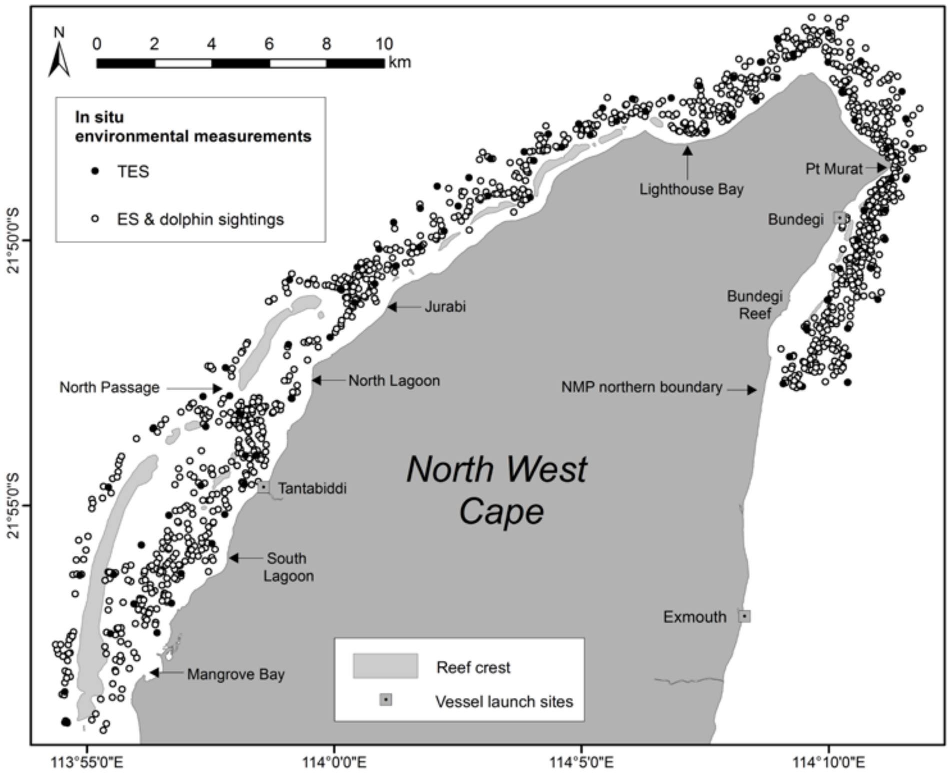

Fig. S1.1. Locations of *in situ* environmental measurements (depth, SST, salinity, turbidity, and pH) collected during boat-based surveys of Australian humpback dolphins in northern Ningaloo Marine Park (NMP) from May 2013 to October 2015. TES (transect environmental station) = 87 fixed locations (black dots) where a mean TES value of each variable was obtained (*n* per TES range 2-30, total *n* = 1582); ES (environmental station) and dolphin sighting data points (white dots, *n* = 1380, includes Indo-Pacific bottlenose dolphin sightings) are variable. Digital environmental layers of water depth (Fig. S1.3a) and SST (Fig. S1.4) were used in ensemble species distribution modelling, but turbidity, salinity, and pH digital layers were omitted from modelling due to low spatial variation and low confidence in predicted values across the study area (data not shown). See Table S1.1 for description of depth and SST layer creation.

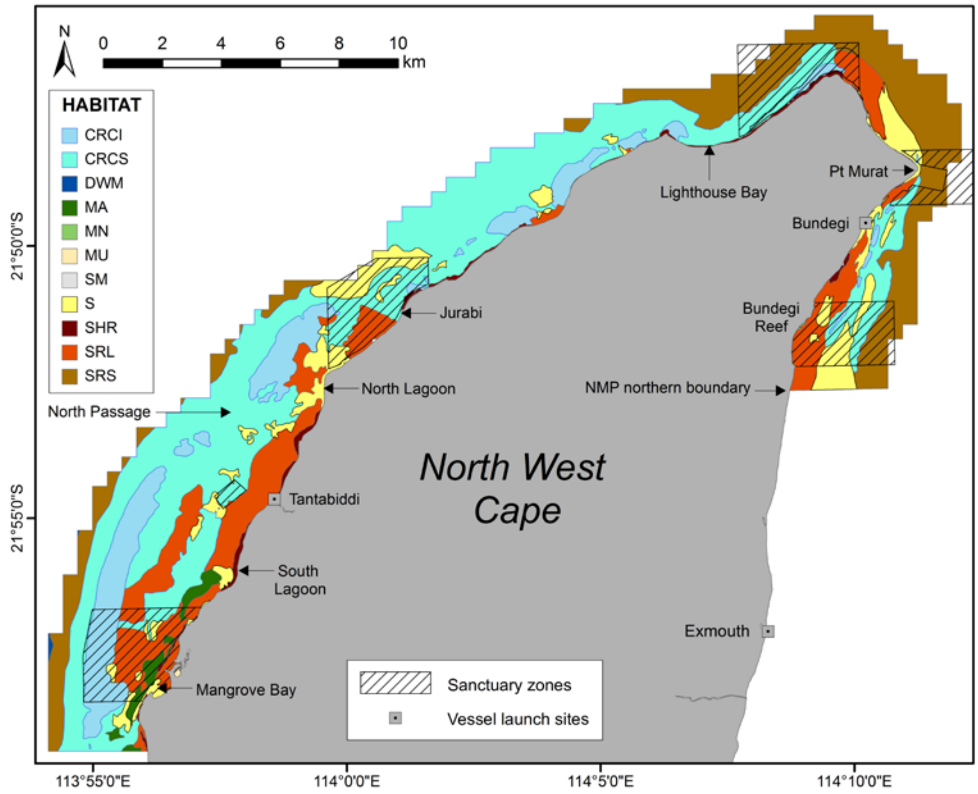

Fig. S1.2. Benthic habitat type (Bancroft and Sheridan 2000) and sanctuary zones of northern Ningaloo Marine Park used in ensemble species distribution modelling of Australian humpback dolphins. CRCI = coral reef communities (intertidal or shallow/limestone); CRCS = coral reef communities (subtidal); S = sand, SRL = subtidal reef (low relief - lagoonal); SRS = subtidal reef (low relief - seaward); SHR = shoreline reef; MA = macroalgae (limestone reef); SM = salt marsh; MN = mangrove, MU = mudflat; and deep DWM = water mixed filter feeding and soft bottom communities. See Table S1.2 for habitat category definitions.

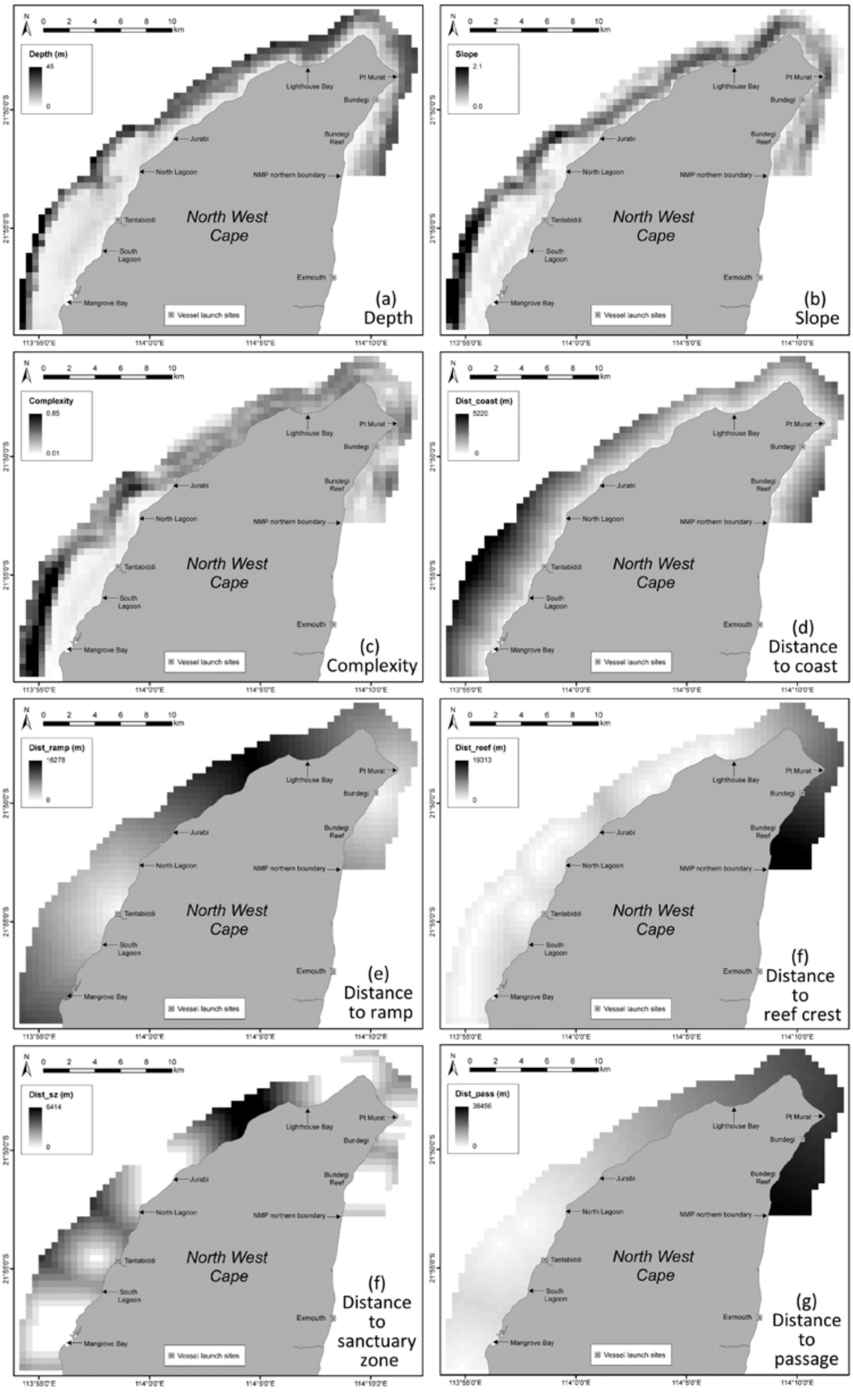

Fig. S1.3. Biotic, abiotic and anthropogenic environmental predictor variables used in ensemble species distribution modelling of Australian humpback dolphins in northern Ningaloo Marine Park (NMP) from May 2013 to October 2015. Layers: (a) depth; (b) slope; (c) seabed complexity; (d) distance to coast; (e) distance to ramp; (f) distance to reef crest; (g) distance to sanctuary zone; (h) distance to passage. Variables sampled at 500 x 500 m grid cell resolution. Raster layers derived in ArcMap 10.3.1 (ESRI). For variable definitions see Table S1.1.

APPENDIX S2

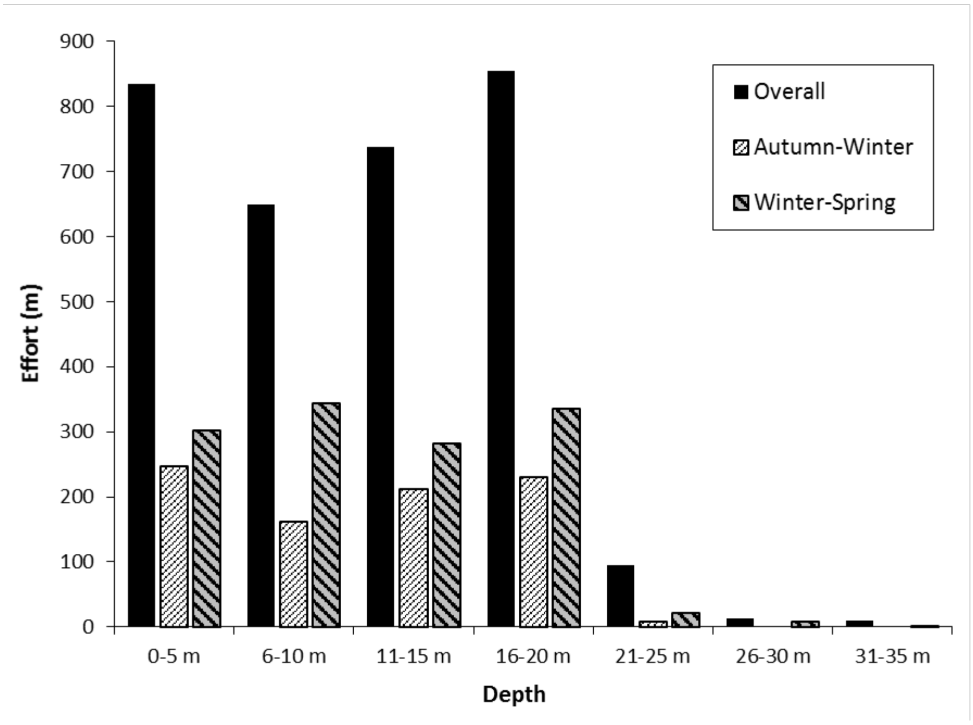

Fig. S2.1. Comparison of survey effort as a function of water depth for boat-based surveys of Australian humpback dolphins in northern Ningaloo Marine Park (NMP) for the overall survey period (May 2013-October 2015), and seasons Autumn-Winter (April-July) and Winter-Spring (August-October). Effort represented as metres of survey track lines per 500 x 500 m grid cell.

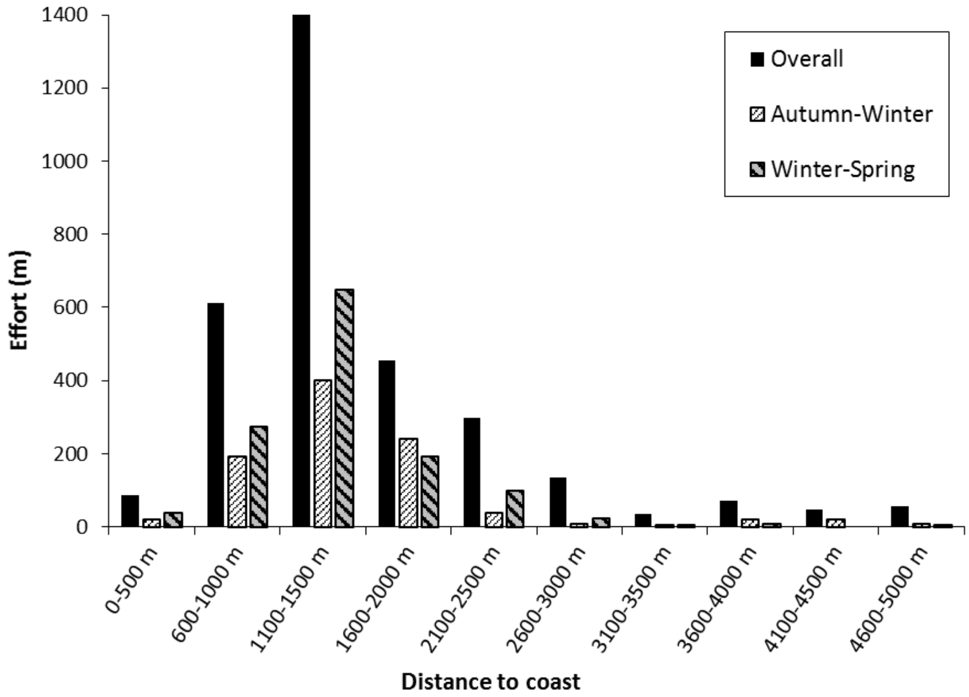

Fig. S2.2. Comparison of survey effort as a function of distance to coast for boat-based surveys of Australian humpback dolphins in northern Ningaloo Marine Park (NMP) for the overall survey period (May 2013-October 2015), and seasons Autumn-Winter (April-July) and Winter-Spring (August-October). Effort represented as metres of survey track lines per 500 x 500 m grid cell.

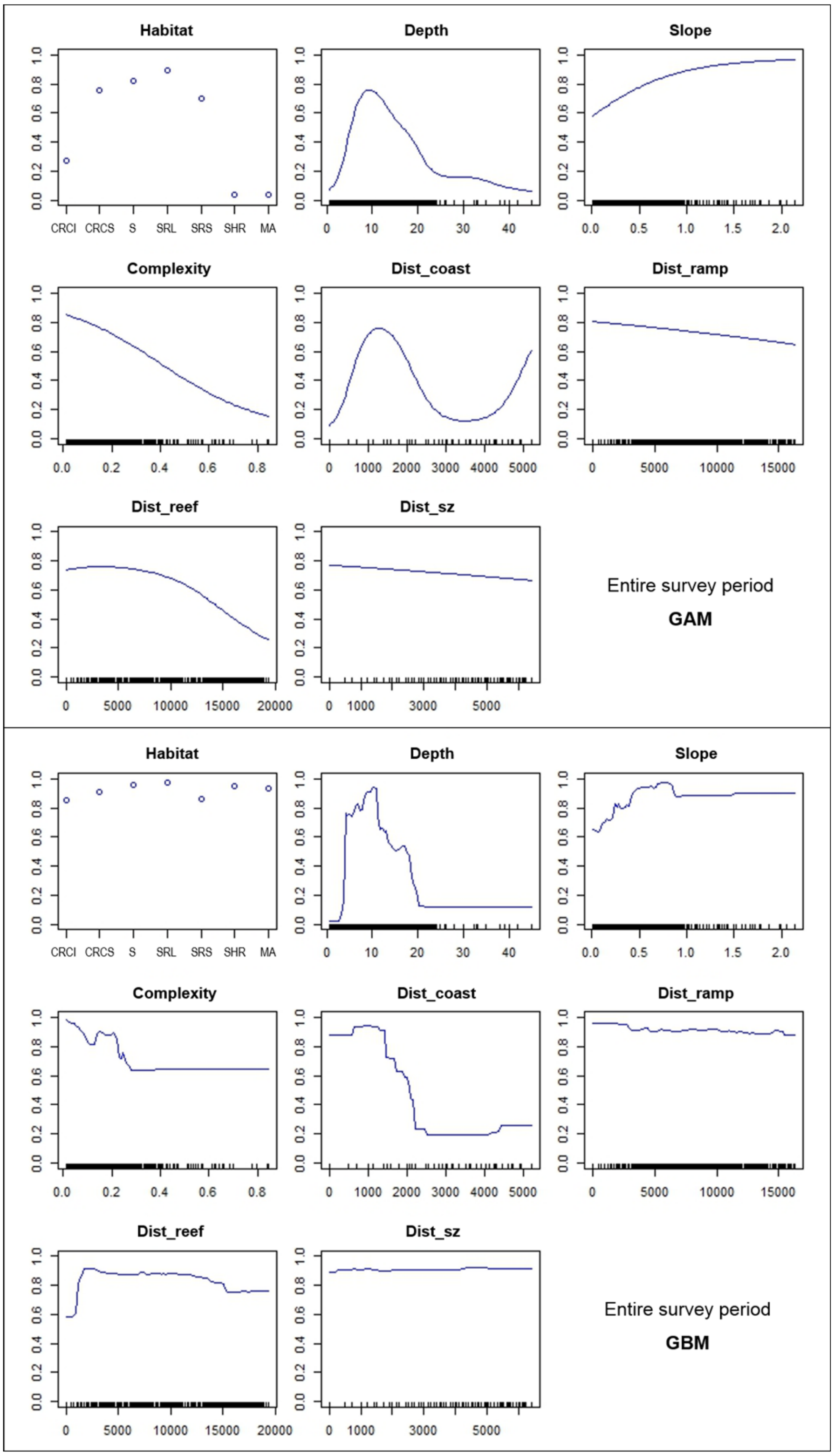
Habitat
Depth
Slope
CRCI CRCS S SRL SRS SHR MA
Complexity
Dist_coast
Dist_ramp
Dist_reef
Dist_sz
Entire survey period
GAM
Habitat
Depth
Slope
CRCI CRCS S SRL SRS SHR MA
Complexity
Dist_coast
Dist_ramp
Dist_reef
Dist_sz
Entire survey period
GBM

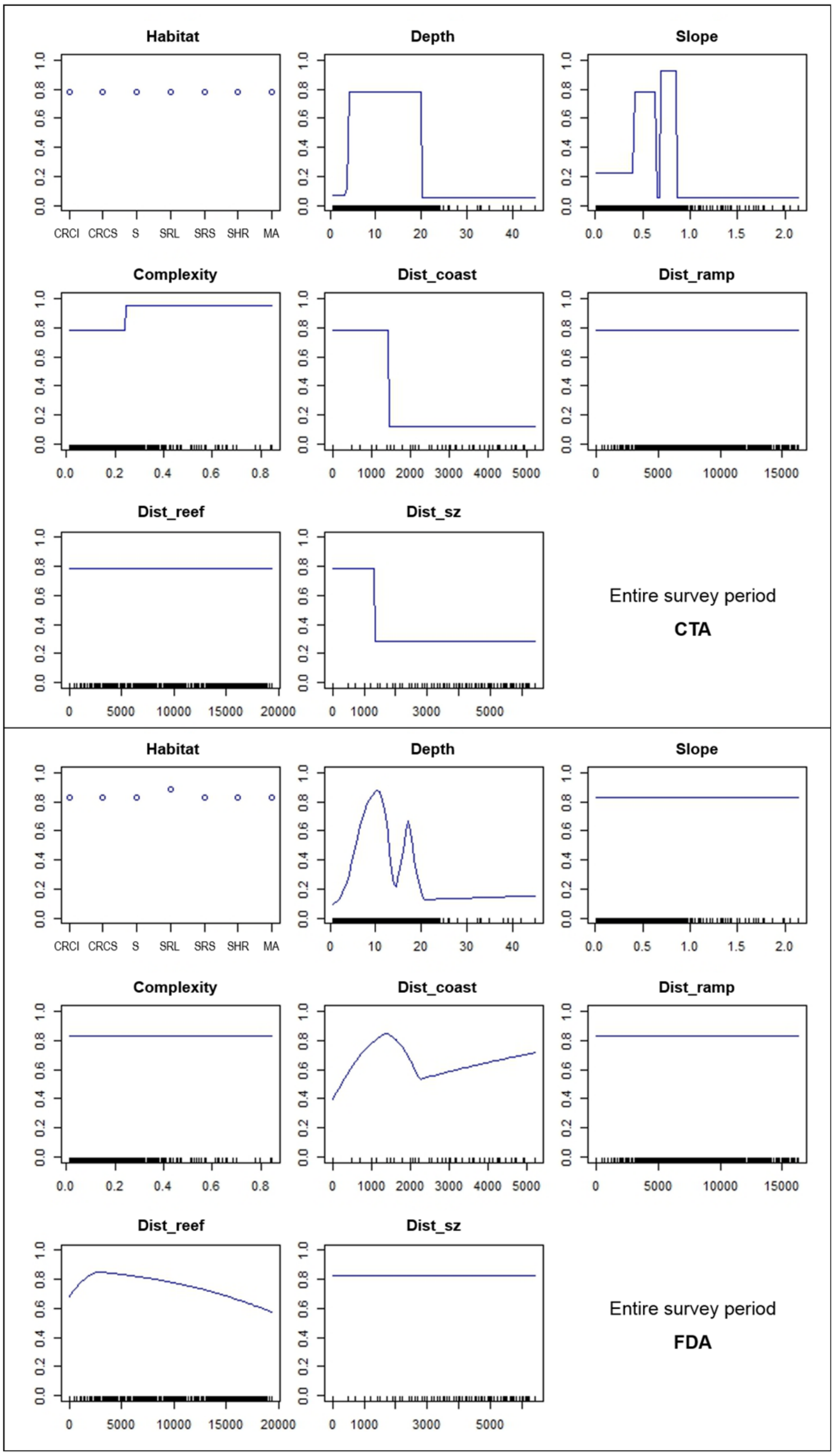
Habitat
Depth
Slope
Complexity
Dist_coast
Dist_ramp
Dist_reef
Dist_sz
Entire survey period
CTA
CRCI CRCS S SRL SRS SHR MA
Habitat
Depth
Slope
Complexity
Dist_coast
Dist_ramp
Dist_reef
Dist_sz
Entire survey period
FDA

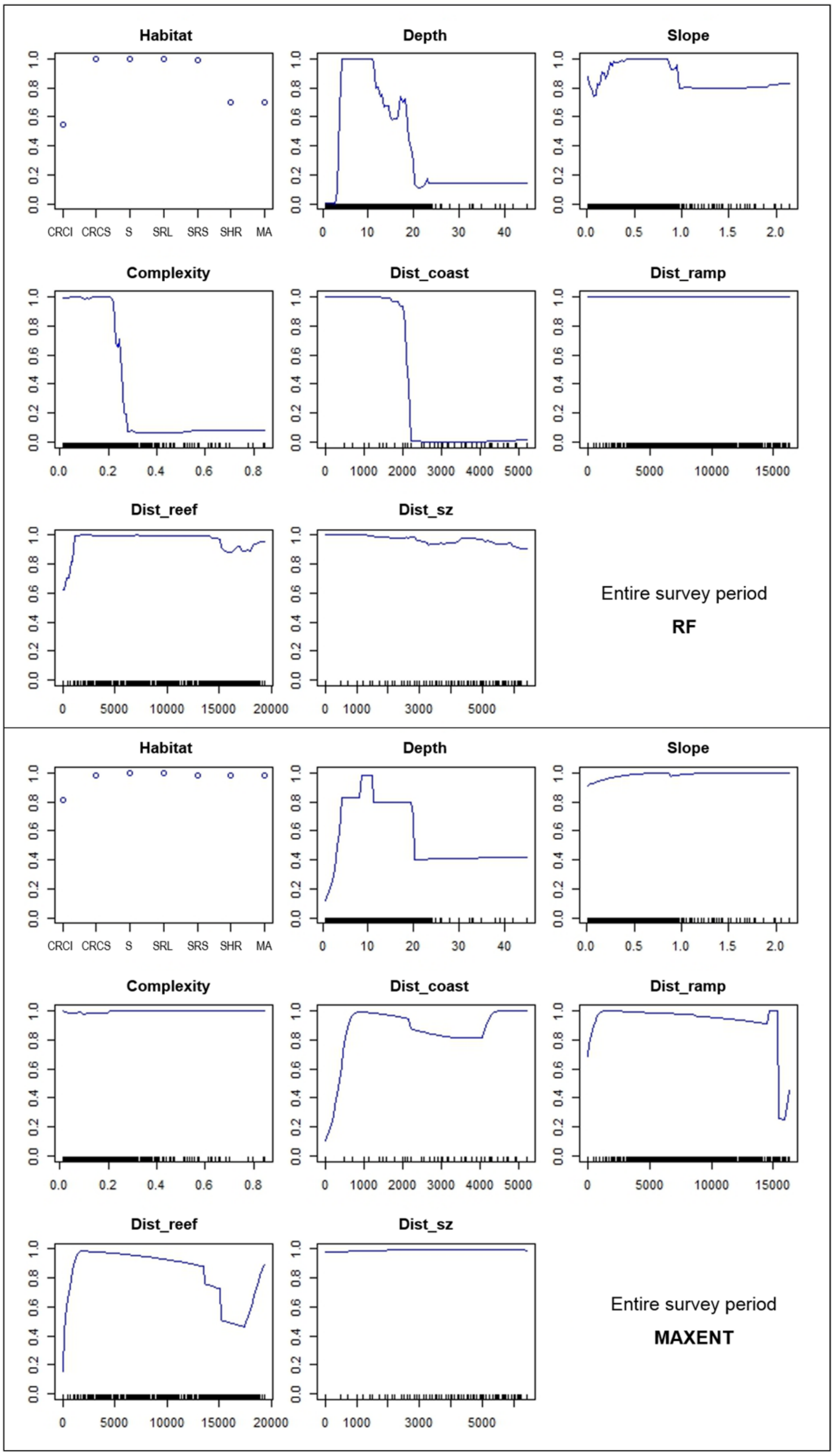

Fig. S2.3. Response curves of presence of Australian humpback dolphins in relation to the environmental predictor variables obtained for species distribution models run for the entire survey period (May 2013 - October 2015) in northern Ningaloo Marine Park. Panes from top to bottom show the curves for each modelling algorithm; GAM = generalised additive model, GBM = generalised boosted model, CTA = classification tree analysis, FDA = flexible discriminant analysis, RF = random forest, and MAXENT = maximum entropy). Each blue line represents the median of the 10 cross-validation runs. For habitat code definitions see Table S1.2 and Figure S1.2.

Model parameters used in biomod2 (extract from R code)

```
-=-=-=-=-=-=-=-=-=-=-=-=-=-=-= 'BIOMOD.Model.Options' -=-=-=-=-=-=-=-=-=-=-=-=-=-=-=-=
GLM = list( type = 'quadratic',
        interaction.level = 0,
        myFormula = NULL,
        test = 'AIC',
        family = binomial(link = 'logit'),
        mustart = 0.5,
        control = glm.control(epsilon = 1e-08, maxit = 50, trace = FALSE
) ),


GBM = list( distribution = 'bernoulli',
        n.trees = 2500,
        interaction.depth = 7,
        n.minobsinnode = 5,
        shrinkage = 0.001,
        bag.fraction = 0.5,
        train.fraction = 1,
        cv.folds = 3,
        keep.data = FALSE,
        verbose = FALSE,
        perf.method = 'cv'),

GAM = list( algo = 'GAM_mgcv',
        type = 's_smoother',
        k = -1,
        interaction.level = 0,
        myFormula = NULL,
        family = binomial(link = 'logit'),
        method = 'GCV.Cp',
        optimizer = c('outer','newton'),
        select = FALSE,
        knots = NULL,
        paraPen = NULL,
        control = list(nthreads = 1, irls.reg = 0, epsilon = 1e-07
```

```
, maxit = 200, trace = FALSE, mgcv.tol = 1e-07, mgcv.half = 15
, rank.tol = 1.49011611938477e-08
, nlm = list(ndigit=7, gradtol=1e-06, stepmax=2, steptol=1e-04, iterlim=200, check.analyticals=0)
, optim = list(factr=1e+07)
, newton = list(conv.tol=1e-06, maxNstep=5, maxSstep=2, maxHalf=30, use.svd=0)
, outerPIsteps = 0, idLinksBases = TRUE, scalePenalty = TRUE, keepData = FALSE
, scale.est = fletcher) ),


CTA = list( method = 'class',
        parms = 'default',
        cost = NULL,
        control = list(xval = 5, minbucket = 5, minsplit = 5, cp = 0.001
, maxdepth = 25) ),


ANN = list( NbCV = 5,
        size = NULL,
        decay = NULL,
        rang = 0.1,
        maxit = 200),

SRE = list( quant = 0.025),

FDA = list( method = 'mars',
        add_args = NULL),

MARS = list( type = 'simple',
         interaction.level = 0,
         myFormula = NULL,
         nk = NULL,
         penalty = 2,
         thresh = 0.001,
         nprune = NULL,
         pmethod = 'backward'),

RF = list( do.classif = TRUE,
       ntree = 500,
```

```
        mtry = 'default',
        nodesize = 5,
        maxnodes = NULL),

MAXENT.Phillips = list( path_to_maxent.jar = 'C:/Workspace/hunt0176/Workspace/R Working Directory/Ss SDM/BIOMOD2/ALL',
            memory_allocated = 512,
            background_data_dir = 'default',
            maximumbackground = 'default',
            maximumiterations = 200,
            visible = FALSE,
            linear = TRUE,
            quadratic = TRUE,
            product = TRUE,
            threshold = TRUE,
            hinge = TRUE,
            lq2lqptthreshold = 80,
            l2lqthreshold = 10,
            hingethreshold = 15,
            beta_threshold = -1,
            beta_categorical = -1,
            beta_lqp = -1,
            beta_hinge = -1,
            betamultiplier = 1,
            defaultprevalence = 0.5),

MAXENT.Tsuruoka = list( l1_regularizer = 0,
                l2_regularizer = 0,
                use_sgd = FALSE,
                set_heldout = 0,
                verbose = FALSE)
-=-=-=-=-=-=-=-=-=-=-=-=-=-=-=-=-=-=-=-=-=-=-=-=-=-=-=-=-=-=-=-=-=-=-=-=-=-=-=-=-=-=-=-=-=-=-=-=-=-=-=-=-=-=
```

APPENDIX S3 – Seasonality of dolphin occurrence

Demographic analysis from Hunt et al. (2017) indicated that there was some seasonality of humpback dolphin movement in and out of the study area. There was however uncertainty regarding the nature of this movement, particularly around temporary emigration rates and apparent survival rates, lagged identification rates and mean residence times, and seasonal abundance estimates having overlapping confidence intervals (see Hunt et al. 2017). The ensemble models showed consistent results in the spatial distribution of humpback dolphins among seasons, and presented herein are the results from the seasonal analysis.

Table S3.1. Summary of survey effort, number of dolphin schools encountered and number of 500 x 500 m grid cells with dolphin presences used to model dolphin distribution within northern Ningaloo Marine Park between May 2013 and October 2015. Autumn-Winter season refers to April to July (inclusive), and Winter-Spring refers to August to October (inclusive).

	Autumn-Winter	Winter-Spring	Total
Survey days (or part thereof)	106	132	238
Survey effort (h)	151	179	330
Survey effort (km)	1,658	1,969	3,627
No. of dolphin schools	73	96	169
No. of grid cells with dolphin presences	62	85	130

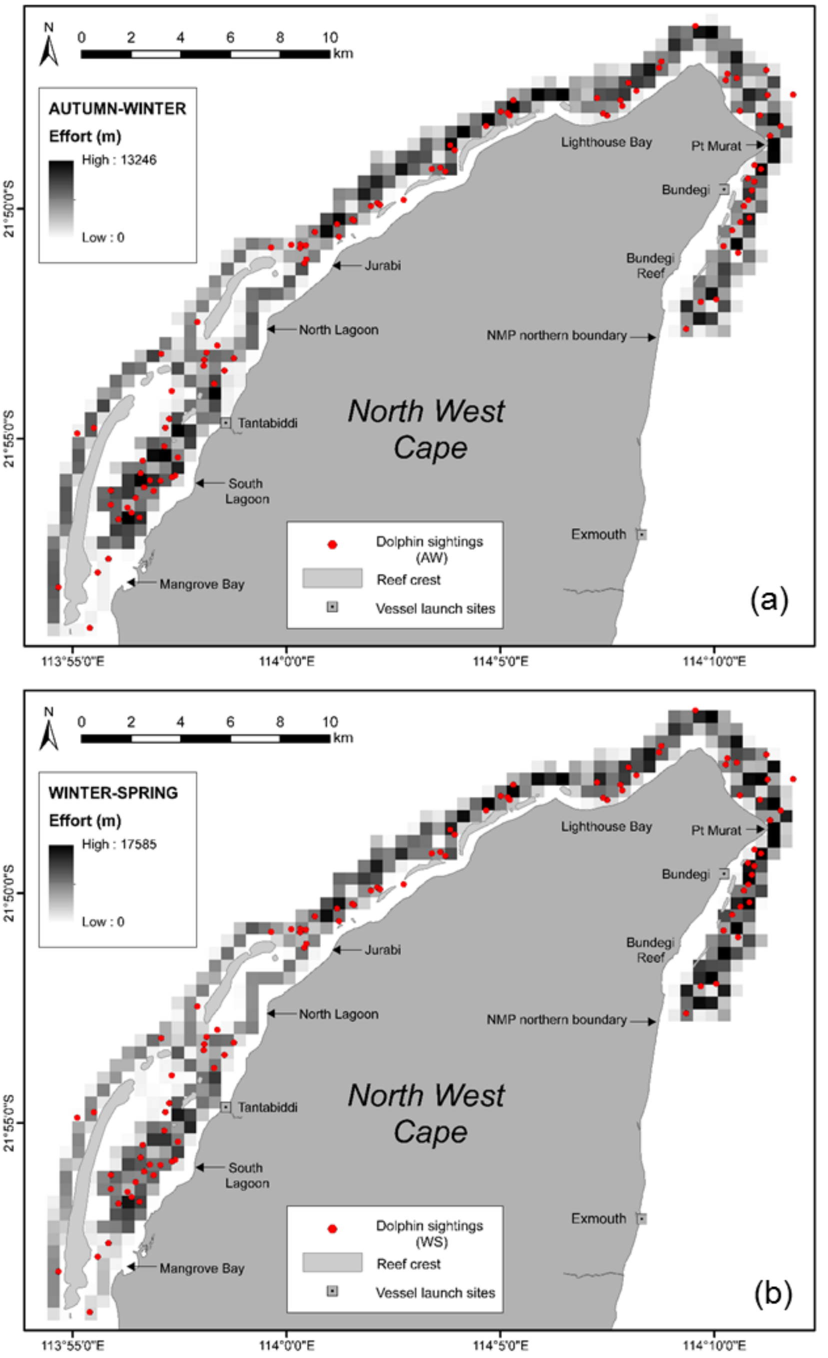

Fig. S3.1. Map of survey effort and sightings of Australian humpback dolphins during boat-based surveys in northern Ningaloo Marine Park (NMP) during the a) Autumn-Winter (AW) period (April-July, n = 73 sightings), and b) Winter-Spring (WS) period (August-October, n = 96 sightings) of May 2013 to October 2015. Effort represented as km of survey track lines per 500 x 500 m grid cell. Dolphin sightings represent single or schools of animals. NB. The survey effort thresholds (m per grid cell) for defining true absences were: 6,739 m for AW (highest was 11,817 m); and 6,778 m for WS (highest was 17,586 m).

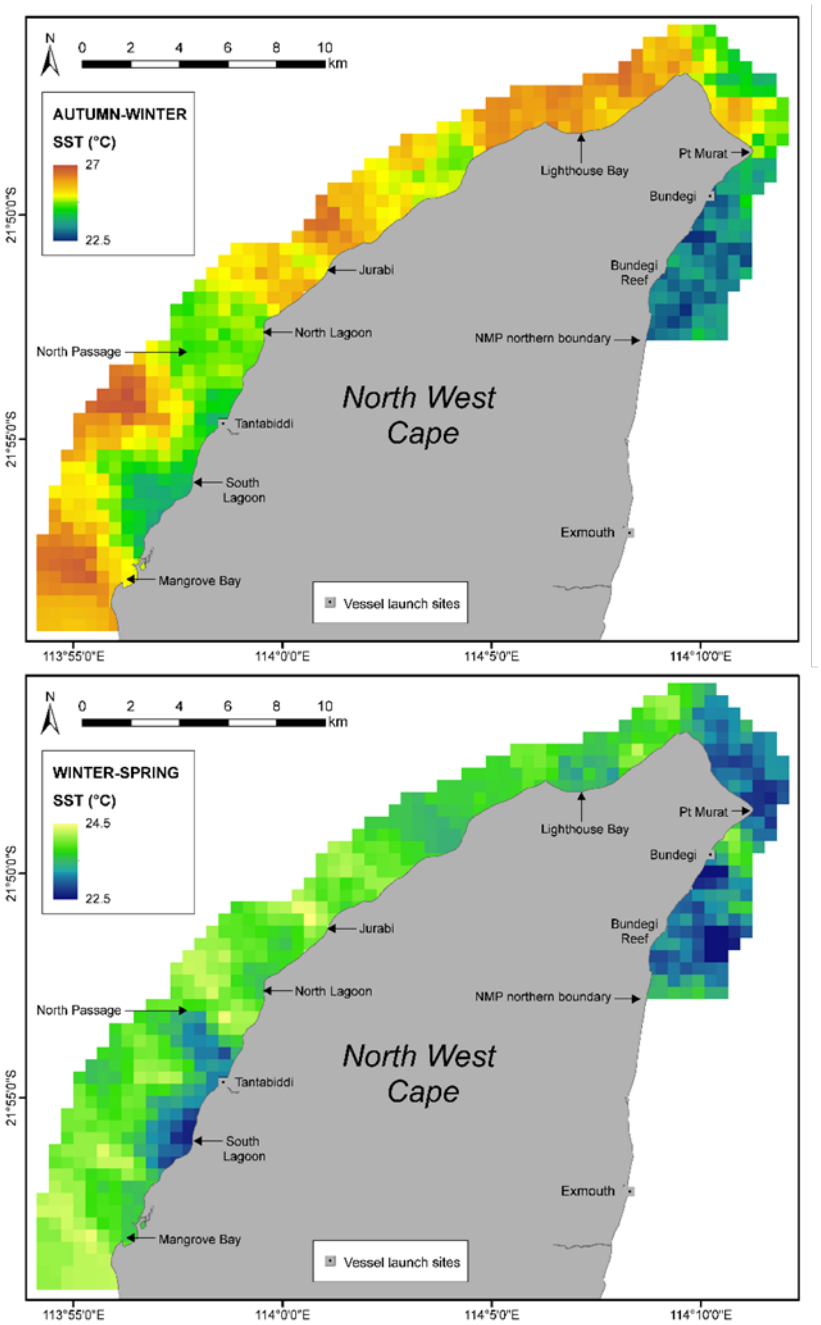

Fig. S3.2. Sea surface temperature (SST) maps of northern Ningaloo Marine Park (NMP) for the Autumn-Winter season (top; April-July) and Winter-Spring season (bottom; August-October) used in ensemble species distribution modelling of Australian humpback dolphins. Layer derived from measurements of data collected *in situ* (see Fig. S2.1) and using the original kriging interpolation in the Spatial Extension of ArcMap 10.3.1 (ESRI). For layer description see Table S1.1.

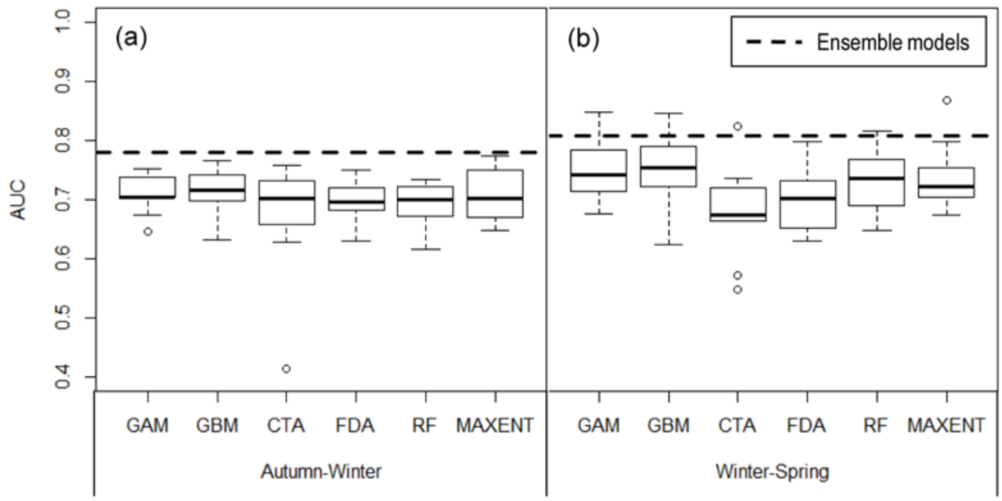

Fig. S3.3. Performance of species distribution models of Australian humpback dolphins in northern Ningaloo Marine Park, Western Australia, built with datasets for the a) Autumn-Winter periods (April-July), and b) Winter-Spring periods (August-October) between May 2013 and October 2015. Box-plot displaying the Area Under Curve (AUC) of the receiver operating characteristics evaluation scores for all models, grouped by modelling algorithm (GAM = generalised additive model, GBM = generalised boosted model, CTA = classification tree analysis, FDA = flexible discriminant analysis, RF = random forest, MAXENT = maximum entropy). Components of box−plot represent minimum (the bottom of the whisker), lower quartile (bottom edge of box), median (bold line drawn inside the box), upper quartile (upper edge of box), maximum (top of the whisker) and outlier AUC values (empty circles) for each modelling method. Dashed line indicates the predictive performance (AUC) of ensemble models for each of the SDM datasets ((AUC was 0.78 for Autumn-Winter and 0.81 for Winter-Spring). Values of AUC ≥ 0.7 indicated that ensemble models performed reasonably well.

Dolphin occurrence across seasons

For the AW and WS datasets, across all individual SDMs (with the exception of WS GAM indicating slope), water depth and distance to coast were again the two most important variables (Table S3.2). This was followed, in general, by slope, seabed complexity, distance to reef crest, and benthic habitat (Table S3.2). Across both seasons, the response curves indicated higher probability of dolphin presence in depths 8-12 m and at a distance less than 2000 m from the coast (Fig. S3.4 & Fig. S3.5). The ensemble model for both seasons indicated a consistent high probability of dolphin occurrence in waters less than 2000 m from the coast, from the NMP northern boundary in the east (i.e. edge of study area), around the NWC to Jurabi in the west (Fig. S3.6). High occurrence was also evident in areas around and inshore of North Passage, and the South Lagoon, but was more prominent in WS than in AW (Fig. S3.6). Dolphin presence was generally higher in shallower waters in WS (mean depth ± SD = 9.2 ± 0.9) than in AW (mean depth ± SD = 12.2 ± 2.6). In AW, dolphin presence was generally higher at a slope less than one (Fig. S3.4), but in WS tended to increase as slope increased (Fig. S3.5). Dolphin presence in AW increased with seabed complexity, but conversely in WS, decreased with an increase in seabed complexity.

Table S3.2. Importance of ecogeographic predictor variables used in species distribution models (SDMs) of Australian humpback dolphins in northern Ningaloo Marine Park over the Autumn-Winter (April-July) period and Winter-Spring (August-October) period of May 2013 to October 2015. Variable importance is presented as the mean over 10 cross-validation runs of each modelling algorithm, and the mean of means amongst them. GAM = generalised additive model, GBM = generalised boosted model, CTA = classification tree analysis, FDA = flexible discriminant analysis, RF = random forest, MAXENT = maximum entropy. Environmental variables of greatest influence based on the randomisation procedure in biomod2 are highlighted in bold. For variable definitions see Table S1.1 in APPENDIX S1.

SDM period	Model	Ecogeographic predictor variables								
		Habitat	Depth	Slope	Complexity	Dist_coast	Dist_ramp	Dist_reef	Dist_sz	SST[a]
Autumn-Winter	GAM	0.278	0.465	0.089	0.105	**0.477**	0.102	0.242	0.074	0.077
	GBM	0.025	**0.356**	0.179	0.032	0.236	0.064	0.149	0.008	0.071
	CTA	0	0.618	0.141	0.094	**0.667**	0.142	0.104	0.024	0.386
	FDA	0.041	**0.777**	0.103	0.000	0.161	0.000	0.081	0.000	0.028
	RF	0.019	**0.206**	0.124	0.055	0.122	0.081	0.097	0.019	0.056
	MAXENT	0.075	**0.405**	0.089	0.04	0.276	0.02	0.073	0.041	0.013
	Mean of means	0.073	**0.471**	0.121	0.054	0.323	0.068	0.124	0.028	0.105
Winter-Spring	GAM	0.215	0.356	**0.401**	0.279	0.389	0.115	0.186	0.063	0.067
	GBM	0.073	**0.499**	0.152	0.204	0.118	0.056	0.062	0.021	0.082
	CTA	0.118	**0.714**	0.154	0.198	0.209	0.117	0.163	0.058	0.213
	FDA	0.064	**0.797**	0.032	0.073	0.135	0.127	0.137	0.083	0.000
	RF	0.049	**0.280**	0.110	0.109	0.086	0.057	0.042	0.024	0.057
	MAXENT	0.059	**0.400**	0.166	0.161	0.215	0.069	0.152	0.040	0.041
	Mean of means	0.096	**0.508**	0.169	0.171	0.192	0.090	0.124	0.048	0.076

[a] Sea surface temperature (SST) is a temporal variable and not included in the entire survey period SDM dataset see Table 3

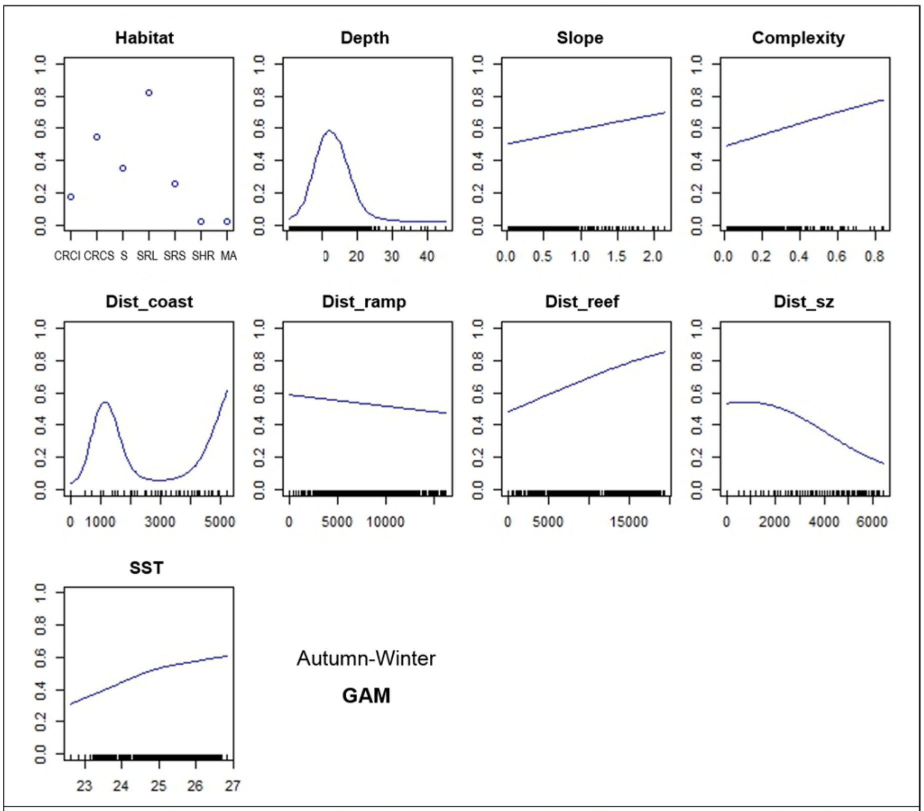
Habitat
Depth
Slope
Complexity
Dist_coast
Dist_ramp
Dist_reef
Dist_sz
SST
CRCI CRCS S SRL SRS SHR MA
Autumn-Winter
GAM

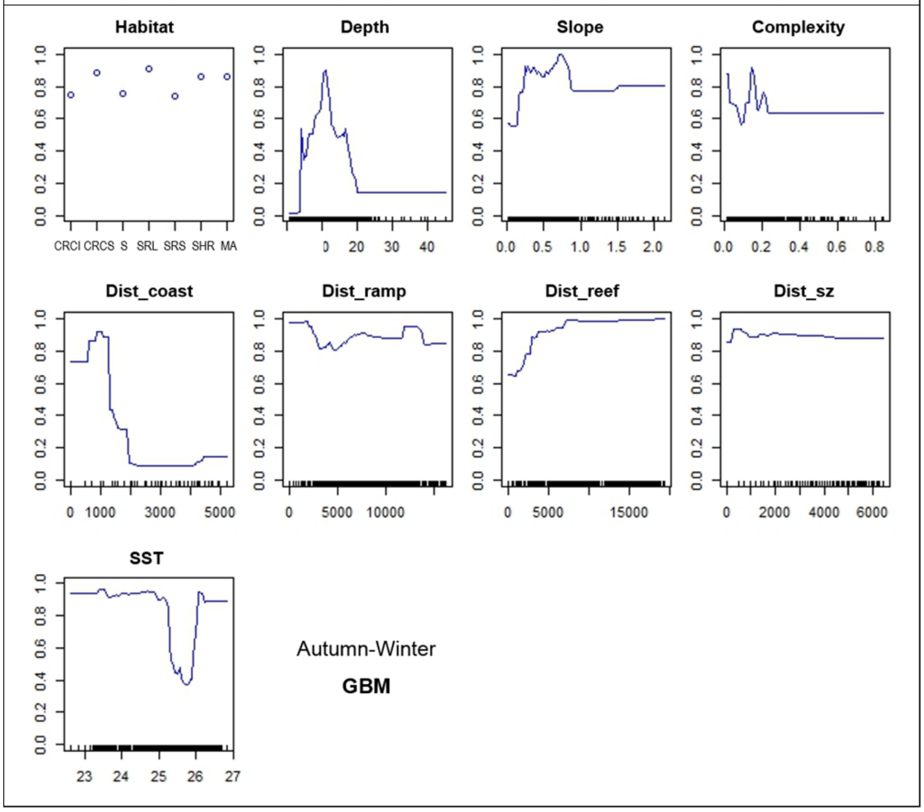
Habitat
Depth
Slope
Complexity
Dist_coast
Dist_ramp
Dist_reef
Dist_sz
SST
CRCI CRCS S SRL SRS SHR MA
Autumn-Winter
GBM

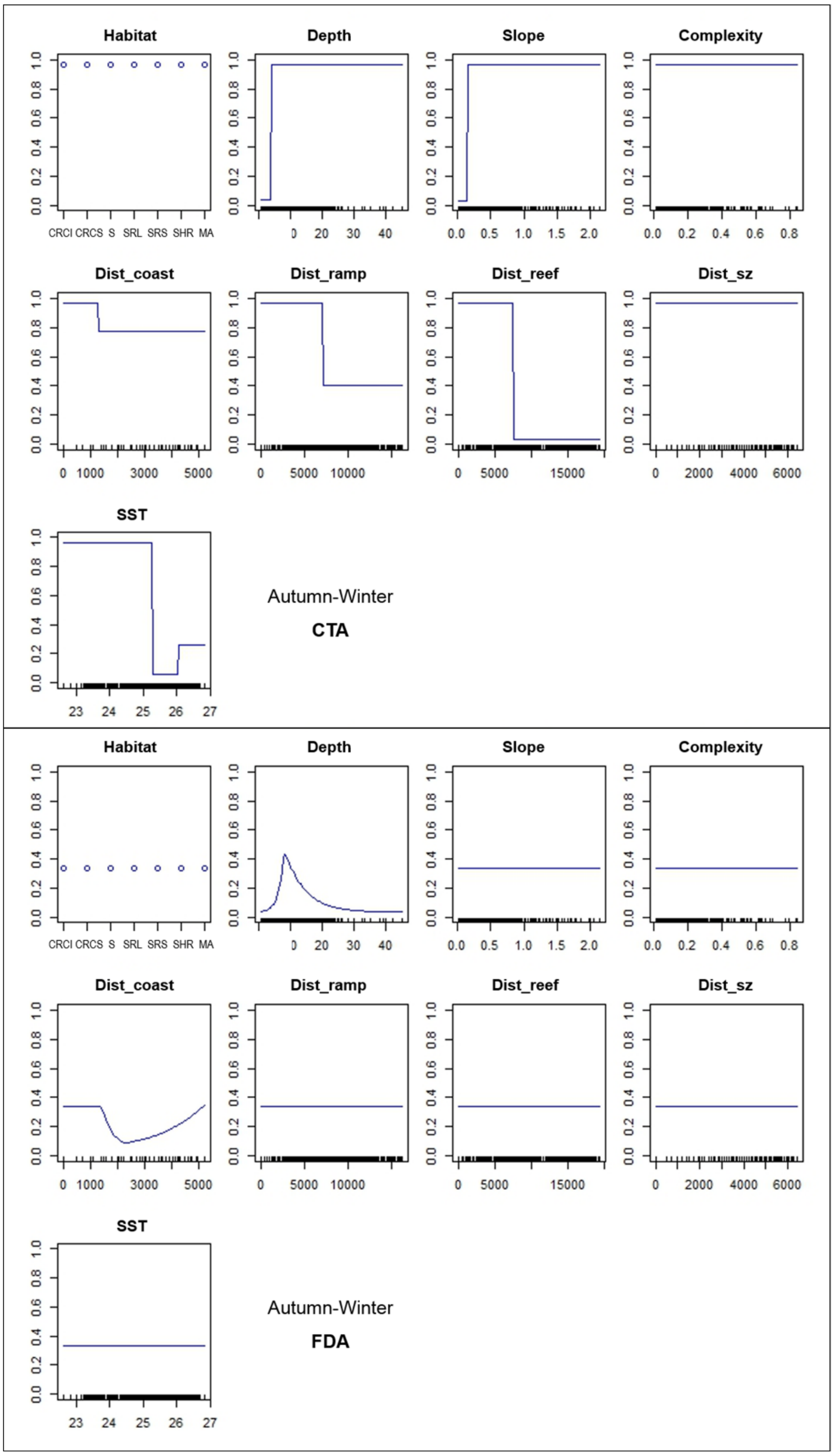
Habitat
Depth
Slope
Complexity
Dist_coast
Dist_ramp
Dist_reef
Dist_sz
SST
CRCI CRCS S SRL SRS SHR MA
Autumn-Winter
CTA
Habitat
Depth
Slope
Complexity
Dist_coast
Dist_ramp
Dist_reef
Dist_sz
SST
CRCI CRCS S SRL SRS SHR MA
Autumn-Winter
FDA

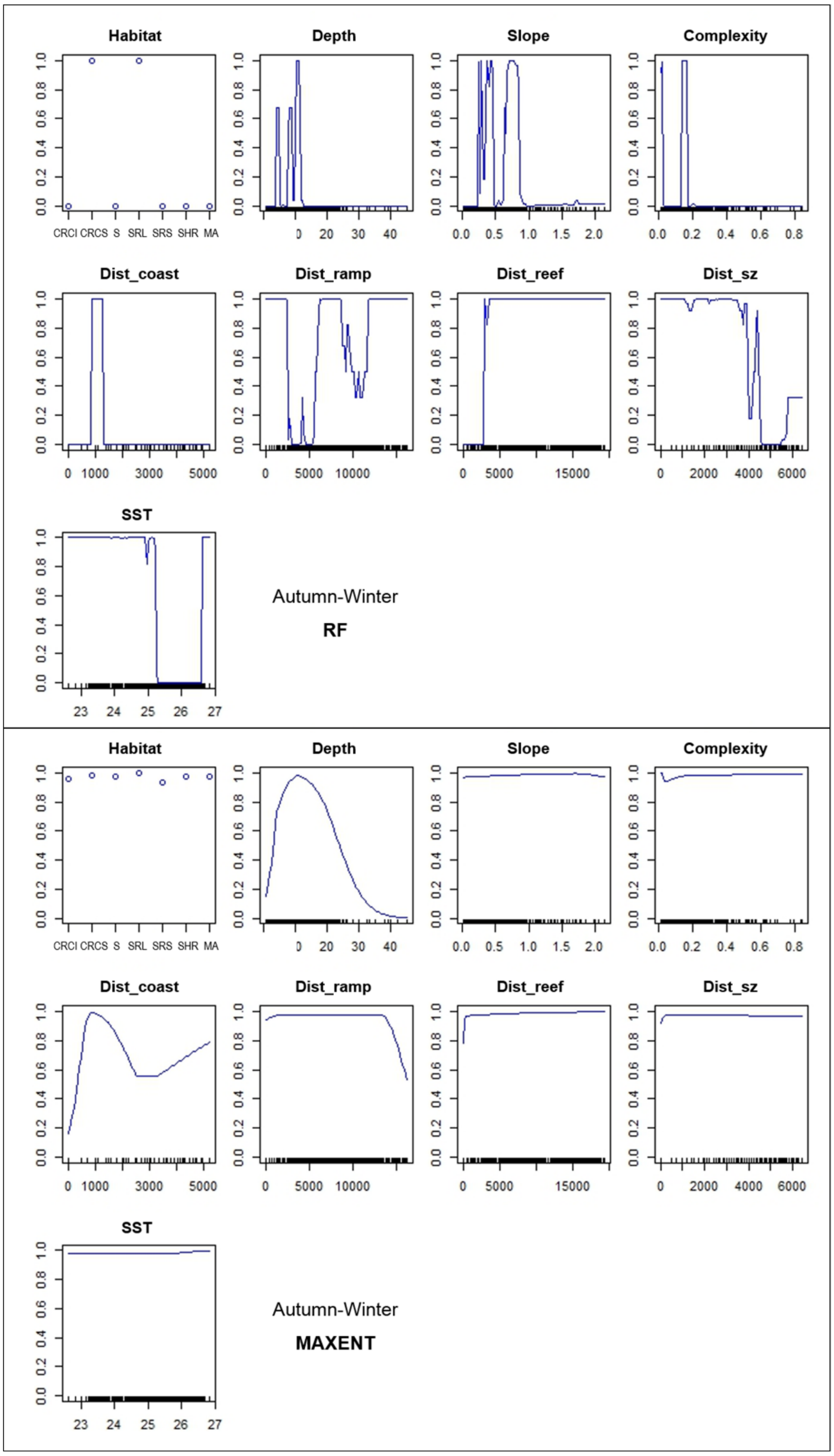

Fig. S3.4. Response curves of presence of Australian humpback dolphins in relation to the environmental predictor variables obtained for species distribution models run for the Autumn-Winter season (April-July) in northern Ningaloo Marine Park. Panes from top to bottom show the curves for each modelling algorithm; GAM = generalised additive model, GBM = generalised boosted model, CTA = classification tree analysis, FDA = flexible discriminant analysis, RF = random forest, and MAXENT = maximum entropy). Each blue line represents the median of the 10 cross-validation runs. For habitat code definitions see Table S2.1 and Figure S1.2.

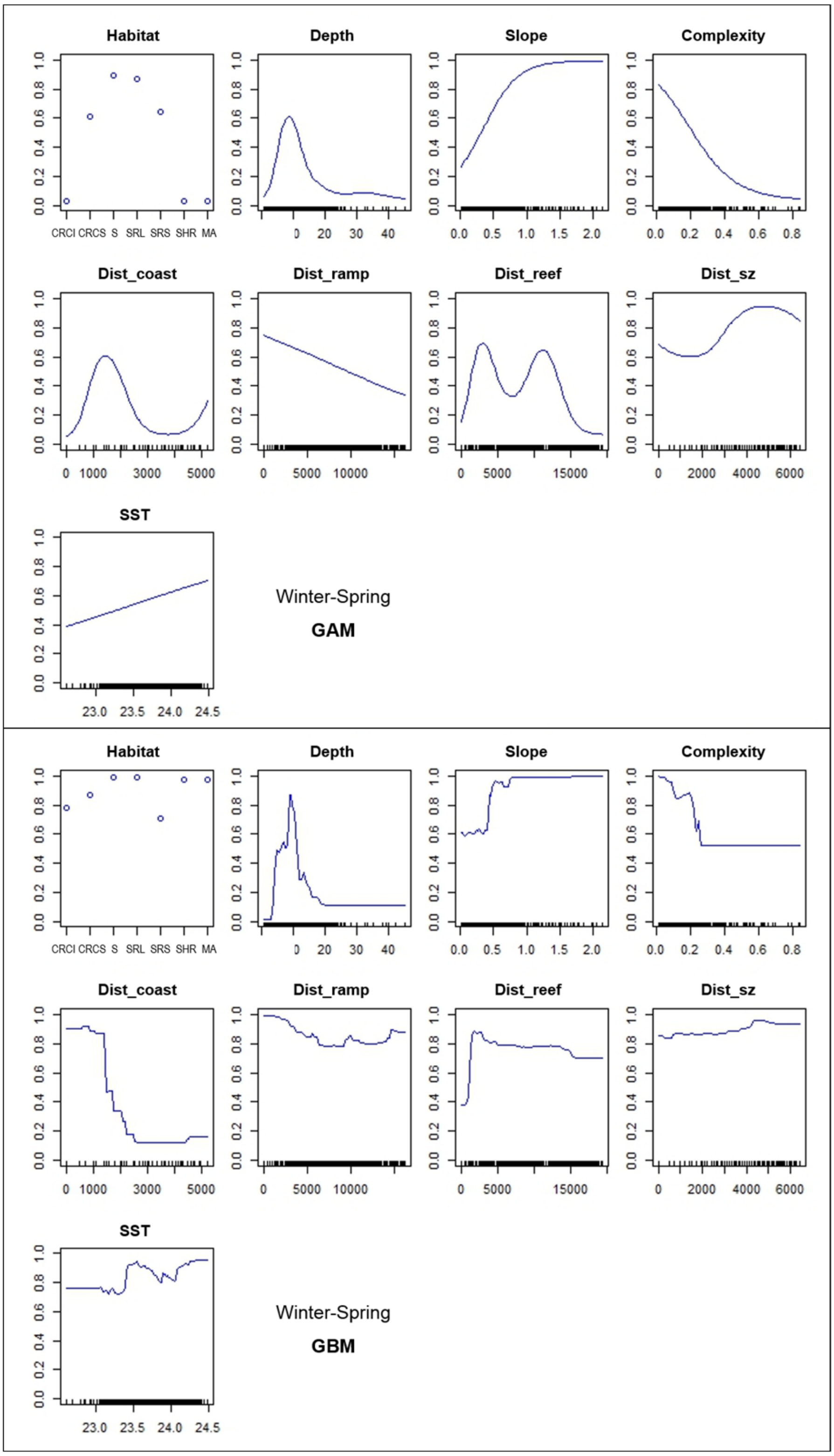
Habitat
Depth
Slope
Complexity
Dist_coast
Dist_ramp
Dist_reef
Dist_sz
SST
CRCI CRCS S SRL SRS SHR MA
Winter-Spring
GAM
Habitat
Depth
Slope
Complexity
Dist_coast
Dist_ramp
Dist_reef
Dist_sz
SST
CRCI CRCS S SRL SRS SHR MA
Winter-Spring
GBM

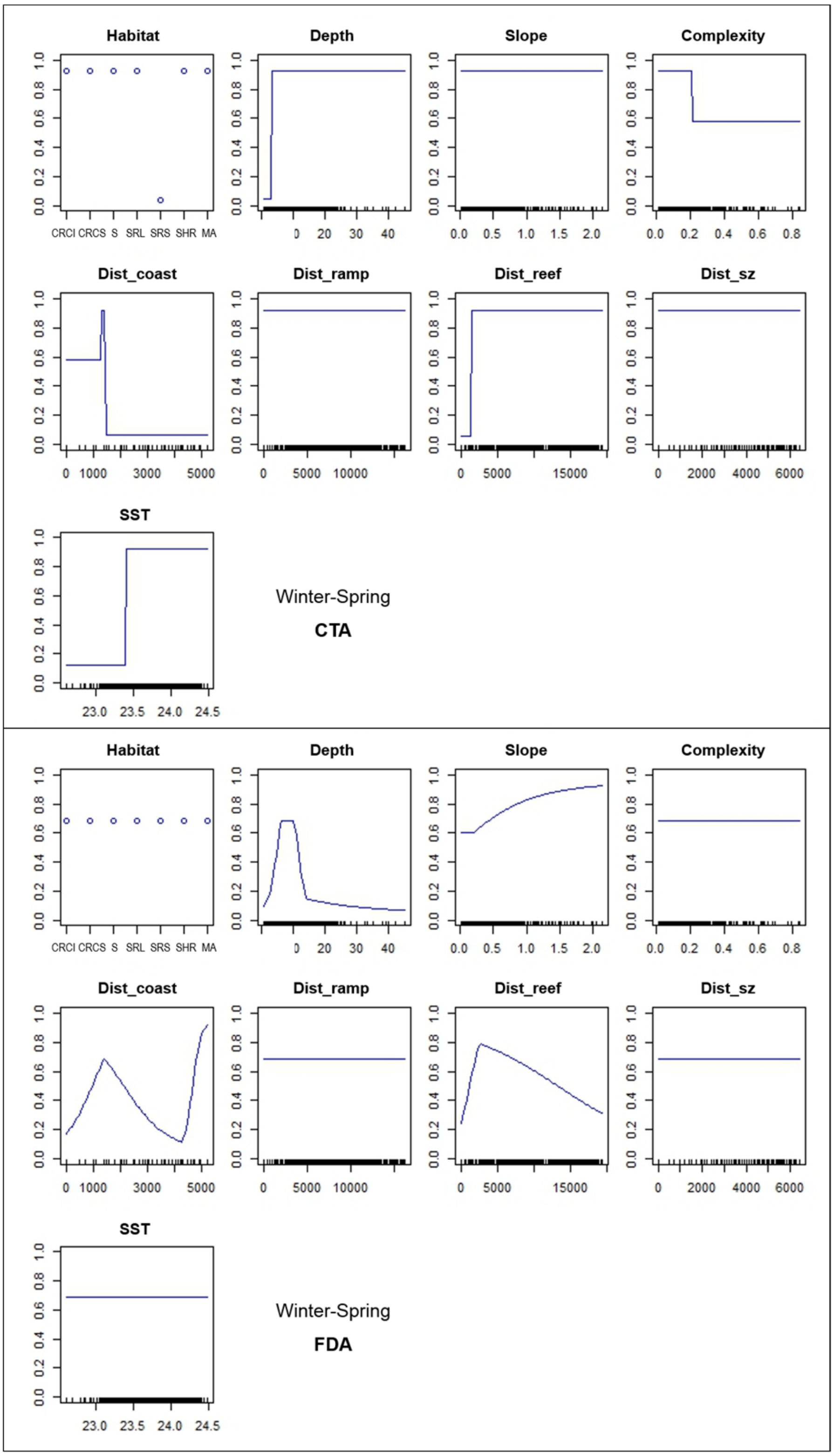
Habitat
Depth
Slope
Complexity
Dist_coast
Dist_ramp
Dist_reef
Dist_sz
SST
CRCI CRCS S SRL SRS SHR MA
Winter-Spring
CTA
Habitat
Depth
Slope
Complexity
Dist_coast
Dist_ramp
Dist_reef
Dist_sz
SST
CRCI CRCS S SRL SRS SHR MA
Winter-Spring
FDA

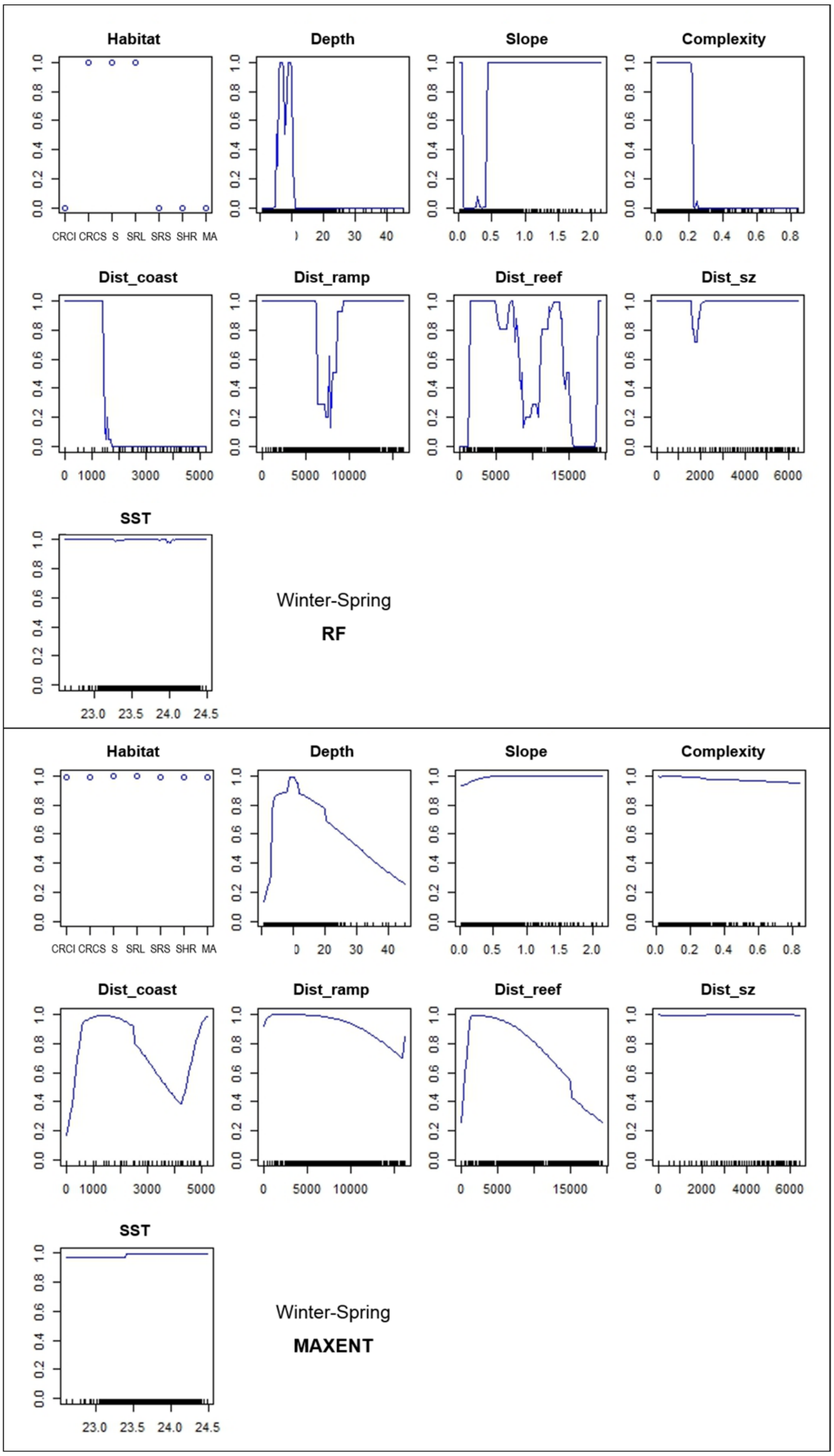

Fig. S3.5. Response curves of presence of Australian humpback dolphins in relation to the environmental predictor variables obtained for species distribution models run for the Winter-Spring season (August to October) in northern Ningaloo Marine Park. Panes from top to bottom show the curves for each modelling algorithm; GAM = generalised additive model, GBM = generalised boosted model, CTA = classification tree analysis, FDA = flexible discriminant analysis, RF = random forest, and MAXENT = maximum entropy). Each blue line represents the median of the 10 cross-validation runs. For habitat code definitions see Table S1.2 and Figure S1.2.

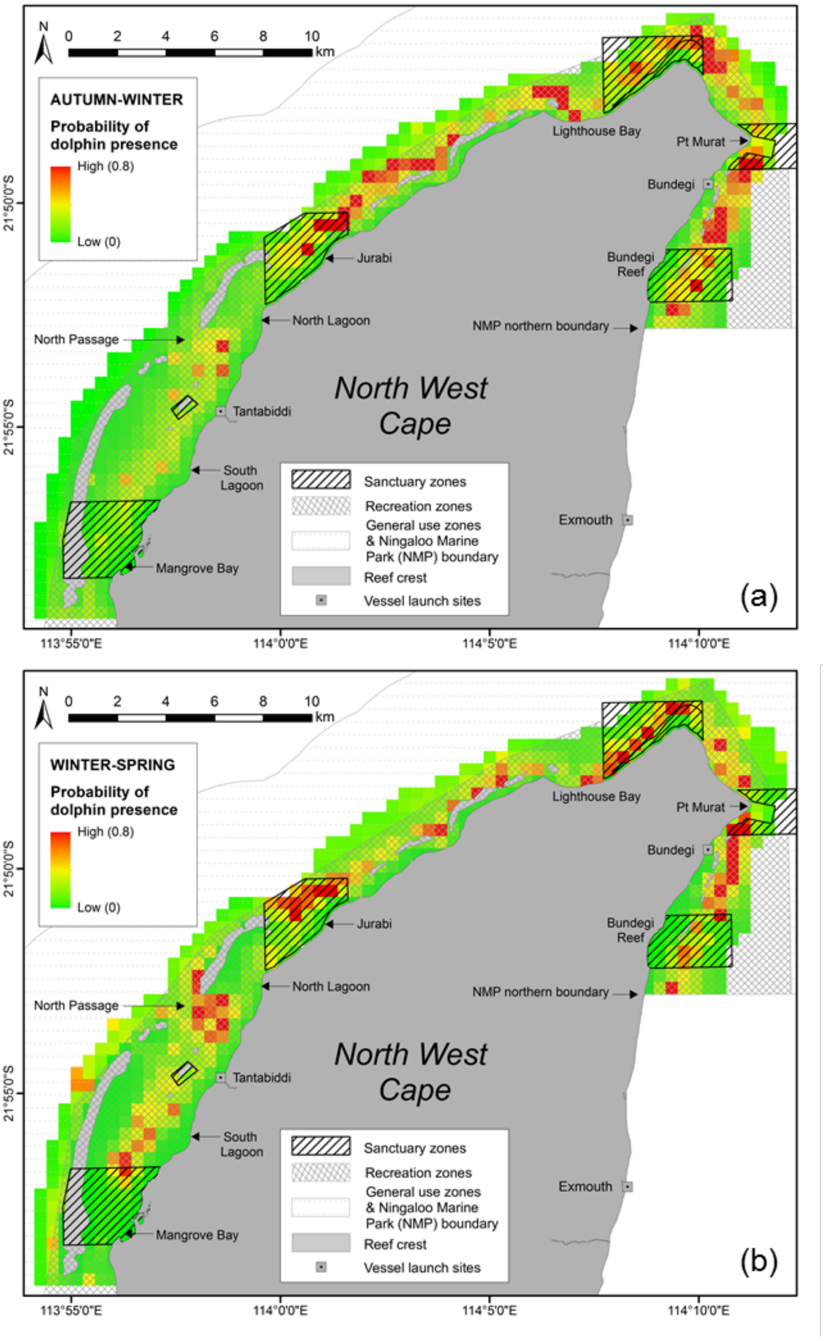

Fig. S3.6. Ensemble model outputs indicating probability of occurrence of Australian humpback dolphins in northern Ningaloo Marine Park (NMP) during the a) Autumn-Winter period (April-July), and b) Winter-Spring period (August-October) of May 2013 to October 2015. Sanctuary zones, recreational zones, general use zones and other locations are also indicated.

Table S3.3. Probability of Australian humpback dolphin occurrence in six sanctuary zones of northern Ningaloo Marine Park predicted by ensemble models for the seasons Autumn-Winter (April-July) and Winter-Spring (August-October) of May 2013 to October 2015. Values shown indicate mean (± SD), median, and range of occurrence probability for the total number of 500 x 500 m grid cells occupying each sanctuary zone, sanctuary zone grids combined, or grids outside sanctuary zones (i.e. recreation and general use zones; RZ and GUZ respectively). See Fig. S3.6 for visual representation of the probability of dolphin occurrence in (and outside) sanctuary zones.

			Dolphin occurrence probability	
Sanctuary zone	Area (km²)	No. of grid cells	*Autumn-Winter* (mean ± SD) (median) (range)	*Winter-Spring* (mean ± SD) (median) (range)
Mangrove Bay	11.4	48	0.06 ± 0.04<br>0.05<br>0.02 - 0.19	0.07 ± 0.10<br>0.04<br>0.03 - 0.55
Tantabiddi	0.5	2	0.12 ± 0.01<br>0.12<br>0.11 - 0.13	0.19 ± 0.07<br>0.14<br>0.14 - 0.24
Jurabi	7.5	36	0.18 ± 0.16<br>0.14<br>0.02 - 0.58	0.26 ± 0.19<br>0.21<br>0.03 - 0.75
Lighthouse Bay	7.6	30	0.18 ± 0.12<br>0.14<br>0.03 - 0.43	0.26 ± 0.21<br>0.20<br>0.03 - 0.74
Point Murat	4.7	9	0.25 ± 0.19<br>0.17<br>0.07 - 0.63	0.25 ± 0.26<br>0.15<br>0.06 - 0.77
Bundegi Reef	7	32	0.17 ± 0.13<br>0.12<br>0.02 - 0.55	0.16 ± 0.16<br>0.07<br>0.03 - 0.70
Combined	38.7	157	0.15 ± 0.13<br>0.10<br>0.02 - 0.63	0.18 ± 0.19<br>0.09<br>0.03 - 0.77
Outside (RZ & GUZ)	111.8	445	0.14 ± 0.13<br>0.09<br>0.02 - 0.70	0.18 ± 0.15<br>0.12<br>0.03-0.80

Table S3.4. Summary of Australian humpback dolphin probability of occurrence throughout the entire study area, and six sanctuary zones in northern Ningaloo Marine Park, for the seasons Autumn-Winter (April-July) and Winter-Spring (August-October) of May 2013 to October 2015. Values shown represent the proportion of low (<0.3), medium (0.31-0.6) and high (>0.6) occurrence probability in each area and temporal period.

Dolphin occurrence probability	*Autumn-Winter*		*Winter-Spring*	
	Entire study area	Sanctuary zones	Entire study area	Sanctuary zones
Low (<0.3)	89%	87%	84%	79%
Medium (0.31-0.6)	10%	11%	13%	16%
High (>0.6)	1%	1%	3%	4%

REFERENCES

Bancroft K, Sheridan M (2000) The major marine habitats of the Ningaloo Marine Park and the proposed southern extension. In. Marine Conservation Branch, Department of Conservation and Land Management, Perth, Western Australia. MMS/PI/NMP&NSE- 26/2000.

DPaW DoF (2014) Ningaloo Marine Park Sanctuary Zones and Murion Islands Management Area Zone Guide. In. Government of Western Australia Department of Parks and Wildlife and Department of Fisheries. http://parks.dpaw.wa.gov.au/park/ningaloo.

Fitzpatrick BM, Harvey ES, Langlois TJ, Babcock R, Twiggs E (2015) Effects of fishing on fish assemblages at the reefscape scale. Marine Ecology Progress Series 524:241-253

Hunt TN, Bejder L, Allen SJ, Rankin RW, Hanf D, Parra GJ (2017) Demographic characteristics of Australian humpback dolphins reveal important habitat toward the southwestern limit of their range. Endangered Species Research 32:71-88

Jefferson TA, Webber MA, Pitman RL (2015) Marine mammals of the world: a comprehensive guide to their identification. Second edition., Vol. Academic Press

Kobryn HT, Wouters K, Beckley LE (2011) Ningaloo Collaboration Cluster: Habitats of the Ningaloo Reef and adjacent coastal areas determined through hyperspectral imagery.

Kobryn HT, Wouters K, Beckley LE, Heege T (2013) Ningaloo reef: shallow marine habitats mapped using a hyperspectral sensor. PLOS ONE 8:e70105

MacLeod CD (2013) An Introduction to Using GIS in Marine Mammal Research. Course Manual. Adelaide, South Australia, 15-19 July 2013 Fremantle, Western Australia, 22-26 July 2013

Parra GJ, Jedensjö M (2014) Stomach contents of Australian snubfin (*Orcaella heinsohni*) and Indo-Pacific humpback dolphins (*Sousa chinensis*). Marine Mammal Science 30:1184-1198

Smallwood CB, Beckley LE, Moore SA (2012) Influence of zoning and habitats on the spatial distribution of recreational activities in a multiple-use marine park. Coastal Management 40:381-400
